# Haplotype‐resolved autotetraploid genome of *Dioscorea nipponica* provides insights into recent polyploidization, metabolic rewiring, and environmental adaptation

**DOI:** 10.1111/tpj.71153

**Published:** 2026-09-28

**Authors:** Shan‐Shan Li, Xiao‐Qin Sun, Yu Feng, Ke Hu, Pan Li, Yu Liu, Jun‐Hao Gu, Min Chen, Rui‐Sen Lu

**Affiliations:** ^1^ Jiangsu Provincial Key Laboratory for Plant Taxonomy, Resource Conservation and Utilization Institute of Botany, Jiangsu Province and Chinese Academy of Sciences Nanjing 210014 China; ^2^ Key Laboratory of Mountain Ecological Rehabilitation and Biological Resource Utilization Chengdu Institute of Biology, Chinese Academy of Sciences (CAS) Chengdu 610041 China; ^3^ College of Life Sciences, Zhejiang University Hangzhou 310058 China

**Keywords:** *Dioscorea nipponica*, autopolyploidy, haplotype‐resolved genome, metabolic trade‐off, population genomics

## Abstract

Whole‐genome duplication (WGD, or polyploidization) is a major evolutionary force in plants, yet the genomic, transcriptional, and metabolic consequences of autopolyploidy remain less understood than those of allopolyploidy. *Dioscorea nipponica*, a medicinal species producing diosgenin‐type steroidal saponins, harbors both diploid and autopolyploid populations, providing a unique system to investigate these processes. Here, we present a haplotype‐resolved, chromosome‐scale genome assembly of autotetraploid *D. nipponica* (2*n* = 4*x* = 40). Comparative and population genomic analyses indicate a single, recent autotetraploid origin (<1.63 Ma), after the divergence of its diploid ancestors (~2.5–2.9 Ma), coinciding with Pliocene–Pleistocene climatic shifts that may have promoted population isolation and genome duplication. The four haplotypes exhibit high average nucleotide identity, extensive synteny and similar global gene expression, with pervasive allele loss (~32% of genes) and widespread allelic transcriptional suppression (~60% of expressed tetrads), together buffering dosage effects. Metabolomic profiling reveals a metabolic trade‐off in rhizomes, with autotetraploids accumulating higher flavonoids but lower diosgenin‐type steroidal saponins than diploids, potentially enhancing reproductive fitness and adaptation to marginal environments. This shift is supported by expanded flavonoid gene families (e.g., PAL, CHS), some exhibiting positive dosage effects, whereas diosgenin pathway genes (e.g., CYP90, CYP94) remain copy number stable and mostly show negative dosage effects in autotetraploids. Selective sweep analysis further identified genes linked to genome stability (e.g., *AtTOP3α*, *AtDMC1*) and abiotic stress response, crucial for polyploid establishment. Overall, these findings offer novel insights into how autopolyploidy shapes gene expression, metabolic evolution, and ecological adaptation, with implications for the conservation and utilization of this medicinally important species.

## Significance Statement

How autopolyploidy reshapes genome architecture, allelic expression, and secondary metabolism remains poorly understood, particularly in medicinal plants. This study presents the first haplotype‐resolved autotetraploid genome of *Dioscorea nipponica*, uncovering its recent evolutionary origin, extensive allelic loss, balanced allelic expression, and metabolic shifts favoring flavonoids over steroidal saponins, offering new insights into polyploid genome evolution and functional consequences.

## INTRODUCTION

Polyploidization, often referred to as whole‐genome duplication (WGD), is a major evolutionary force driving speciation, adaptation, and ecological success in angiosperms (Soltis et al., 2014; Van de Peer et al., 2017). Approximately 30–35% of extant flowering plants are polyploid, underscoring the widespread impact of WGD on plant evolution (Jiao et al., 2011; Soltis et al., 2010; Wood et al., 2009). Based on chromosome origin, polyploids are generally classified as autopolyploids, arising through genome duplication within a single species, and allopolyploids, originating from interspecific hybridization followed by genome doubling (Soltis et al., 2010; Van de Peer et al., 2021). Historically, autopolyploids were regarded as far less prevalent than allopolyploids and were often assumed to suffer greater intrinsic disadvantages, leading to their relative neglect in evolutionary and genomic studies (Barker et al., 2016; Soltis et al., 2007; Spoelhof et al., 2017). However, large‐scale chromosomal surveys have revealed that autopolyploids are widespread across angiosperms and frequently represent the dominant polyploid cytotypes within species, implying that their contribution to plant evolution has been systematically underestimated (Kolář et al., 2017; Rice et al., 2015).

Beyond their frequency in nature, autopolyploids often exhibit pronounced phenotypic and physiological shifts relative to their diploid progenitors, including altered secondary metabolism and enhanced tolerance to abiotic stresses such as cold, drought, and salinity (Conant, 2014; Te Beest et al., 2012; Visger et al., 2016). One widely proposed mechanism underlying these traits is gene dosage effect. Owing to high chromosomal homology and reduced regulatory conflict, autopolyploids can more efficiently translate increased gene copy numbers into elevated expression of dosage‐sensitive genes involved in key metabolic and stress‐response pathways (Bomblies, 2020; Chen, 2007; Qiu et al., 2020; Yoo et al., 2014). For example, in *Cyclocarya paliurus*, WGD and associated dosage effects likely contribute to ecological evolution through enhanced photosynthesis and increased accumulation of triterpenoids (Qu et al., 2023), whereas in *Rheum officinale*, autotetraploidy results in the expansion and transcriptional upregulation of genes involved in the anthraquinone biosynthetic pathway, collectively enhancing stress tolerance and ecological performance (Zhang, He, et al., 2024).

Nevertheless, increased gene dosage alone cannot fully account for the ecological and metabolic consequences of polyploidization. Polyploidization often triggers complex genomic and regulatory reorganization, including biased gene retention or loss, sub‐ or neofunctionalization of duplicates, and allele‐specific expression (Gaynor et al., 2020; Zhang, Shi, et al., 2024). These processes can reshape metabolic networks in a pathway‐ or branch‐specific manner, producing heterogeneous shifts in secondary metabolites rather than a uniform increase (Conant, 2014; Li et al., 2016; Segraves, 2017). For instance, in tetraploid *Solanum commersonii*, phenylpropanoids are generally more abundant than in diploids, whereas major glycoalkaloids accumulate preferentially in diploids (Caruso et al., 2011). Given the roles of phenylpropanoids in pathogen defense and abiotic stress responses, such differential metabolic reallocation likely confers adaptive advantages under specific environmental conditions (Caruso et al., 2011; Dong & Lin, 2021). Despite these observations, how polyploidy reprograms metabolism across genomic, transcriptional, and metabolic levels, and whether such changes confer broad fitness advantages or instead facilitate context‐dependent adaptation, remains largely unexplored (Fasano et al., 2016; Gaynor et al., 2020).

*Dioscorea nipponica* Makino, a perennial herb belonging to the section *Stenophora* in the genus *Dioscorea* (Dioscoreaceae), is widely distributed across the Sino‐Japanese floristic region (Hu et al., 2023; Obidiegwu et al., 2020). The dried rhizome of *D. nipponica*, commonly known as *chuan shan long*, is an important traditional Chinese medicine documented in the Pharmacopeia of the People's Republic of China (2020 edition). It has long been used to treat rheumatic pain, cough, and asthma, and modern pharmacological studies have confirmed its diverse bioactivities, including analgesic, antitussive, anti‐inflammatory, and cardiovascular‐protective effects (Bandopadhyay et al., 2022; Ou‐Yang et al., 2018). These medicinal properties are largely attributed to diosgenin‐type steroidal saponins, with the aglycone diosgenin serving as a vital natural precursor for the semi‐synthesis of steroidal drugs (Mandal et al., 2025). Traditionally, *D. nipponica* was subdivided into two subspecies based on chromosome number (2*n* = 20 versus 40), rhizome bark characteristics, and geographic distribution (Ding & Gilbert, 2000). Our recent genomic surveys, however, have uncovered a more complex intraspecific ploidy series in natural populations, comprising diploid (2*x*), tetraploid (4*x*), hexaploid (6*x*), and octoploid (8*x*) cytotypes, with polyploids inferred to be autopolyploids (Hu et al., 2025). Notably, autopolyploids are predominantly distributed in peripheral regions of the species' range, often characterized by harsh environments such as high altitudes, high latitudes, or cold climates, while diploids are more common in central regions (Chen, 2007; Hu et al., 2025), suggesting that autopolyploidy may enhance adaptive capacity and ecological success.

Chromosome‐scale genome assemblies and diosgenin biosynthetic pathways have been well characterized in diploid members of section *Stenophora*, including diploid *D. nipponica* (Hu et al., 2025; Wang et al., 2026) and its closely related species *Dioscorea zingiberensis* (Cheng et al., 2021; Li et al., 2022), whereas recent studies have also begun to investigate diosgenin biosynthesis and regulation in polyploid *Dioscorea* species, such as autotetraploid *Dioscorea polystachya* from section *Enantiophyllum* (Shan et al., 2026). However, these studies have primarily focused on diosgenin biosynthesis within individual species, with limited efforts to systematically compare diploid and autopolyploid cytotypes within the same species under comparable genetic backgrounds, thereby constraining our understanding of the direct consequences of genome duplication. In particular, it remains unclear whether autopolyploidy leads to a general increase in secondary metabolites, including diosgenin‐derived steroidal saponins, or instead drives metabolic reallocation toward specific compound classes, such as flavonoids or phenylpropanoids, that may contribute to stress adaptation (Caruso et al., 2011). Moreover, the genomic and transcriptomic reorganizations underlying these potential metabolic shifts, including gene retention and loss, allele‐specific expression and dosage effects, as well as the demographic origins and adaptive mechanisms of natural autopolyploid populations, also remain unclear.

To address these questions, we generated a chromosome‐scale, haplotype‐resolved genome assembly of autotetraploid *D. nipponica* and integrated transcriptomic, metabolomic, and population genomic analyses to investigate: (1) how WGD reshapes gene expression and secondary metabolism, particularly diosgenin biosynthesis and potential trade‐offs with other metabolites; (2) the demographic origins of natural autotetraploid populations; and (3) the genetic basis of their environmental adaptation. Together, these results will provide novel insights into the evolutionary and functional consequences of autopolyploidy, with important implications for the conservation, utilization, and breeding of this medicinally important species.

## RESULTS

### Genome sequencing, ploidy inference, and chromosome‐scale assembly

The genome size of tetraploid *D. nipponica* (2*n* = 4*x* = 40) was estimated to be ~2.22 Gb (2C) based on flow cytometry (Figure S1), and 2.03 Gb using *k*‐mer distribution analysis (Figure 1a). These estimates corresponded to a monoploid genome size of ~500 Mb and were consistent with previously reported diploid genomes of *D. nipponica* and *D. zingiberensis*. The repeat content of tetraploid *D. nipponica* was estimated at 50.98%, and the heterozygosity level was relatively high (~3.12%), which likely facilitated the separation of the four haplotypes during haplotype‐resolved assembly. To determine whether tetraploid *D. nipponica* was autotetraploid or allotetraploid, three complementary approaches were employed. GenomeScope profiling revealed a strong predominance of homozygous *k*‐mers (aaaa, 96.88%), followed by aaab (2.13%) and aabb (0.84%), a pattern consistent with expectations for an autotetraploid genome, in which aaab configurations are expected to exceed aabb (Figure S2). Smudgeplot results further identified a dominant signal at a minor allele ratio (B/[A + B]) of ~0.25, with the corresponding total *k*‐mer coverage (A + B) centered around four times the monoploid coverage, consistent with a tetraploid genome. Notably, AAAB configurations were markedly more abundant than AABB, further supporting an autotetraploid genome structure (Figure 1b). Consistently, nQuire analysis revealed characteristic base frequency peaks at ~0.25, 0.5, and 0.75 across variable sites, providing convergent evidence that tetraploid *D. nipponica* is of autopolyploid origin (Figure 1c).

**Figure 1:**
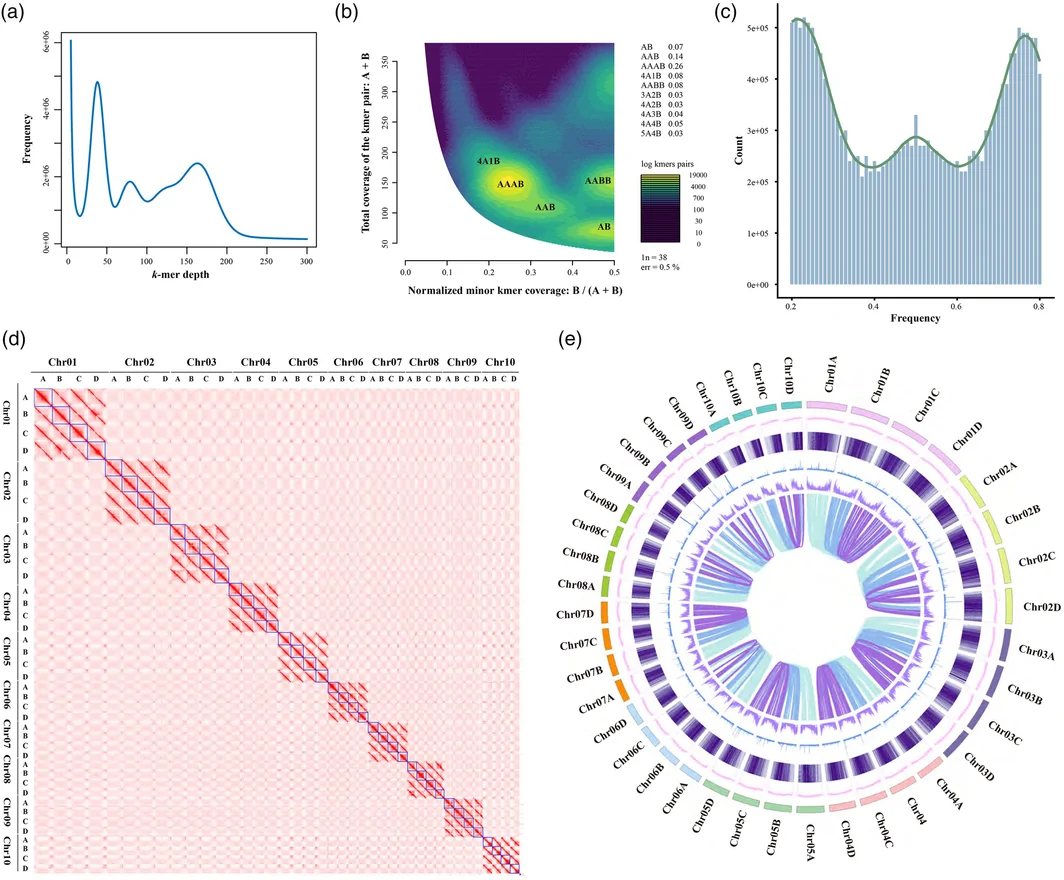
Genomic characteristics and overview of the autotetraploid *Dioscorea nipponica* genome. (a) *K*‐mer based genome size estimates of autotetraploid *D. nipponica* using *k* = 19. A total of 77 168 550 280 19‐mers were detected with a peak depth of 38, corresponding to an estimated 2C genome size of 2.03 Gb (calculated as total *k*‐mer number/peak depth). (b) Smudgeplot analysis of autotetraploid *D. nipponica*. (c) Distribution of base frequencies at variable sites inferred by nQuire, showing three peaks at 0.25, 0.50, and 0.75, consistent with an autotetraploid genome. (d) Hi‐C contact map of the autotetraploid *D. nipponica* genome. (e) Genomic features of autotetraploid *D. nipponica*. The Circos plot displays the 40 chromosomes (outer ring) and concentric tracks (from inside out): synteny links, gene density, tandem repeat density, transposable element (TE) density (heatmap), and GC content.

A total of 84.67 Gb (38.14×) PacBio HiFi reads were assembled using hifiasm with Hi‐C integration (Table S1). The initial assembly produced a primary assembly of 1.19 Gb (1364 contigs; contig N50 = 24.73 Mb) and two phased haplotype assemblies (hap1 = 1.21 Gb, hap2 = 0.87 Gb; Table S2). The combined length of the phased haplotypes (2.08 Gb) closely matches the independently estimated tetraploid genome size (2C = 2.03–2.22 Gb; Figure 1a; Figure S1). To achieve chromosome‐scale resolution, the two phased haplotypes were merged and processed using CPhasing (‐n 10:4) with 257.00 Gb Hi‐C data (115.77× coverage; Table S3). This resulted in the partitioning of the assembly into 10 homologous chromosome groups corresponding to the basic chromosome number (*x* = 10), and subsequent resolution of each group into four haplotypes (A, B, C, and D), yielding 40 pseudochromosomes (Figure 1d,e; Table S4). In total, 1.96 Gb of non‐redundant genomic sequences were successfully anchored to chromosomes, representing the chromosome‐scale tetraploid assembly (Figure 1e; Table S4). Chromosome lengths ranged from 34.84 to 73.65 Mb, with the largest chromosomes (Chr01A–Chr01D) spanning 70.57–73.65 Mb and the smallest chromosomes (Chr10A–Chr10D) measuring approximately 35.14–37.27 Mb (Table S4). The proportion of gap sequences (Ns) across chromosomes was extremely low (0–0.01% of chromosome length), with only 1–26 scaffold gaps per chromosome and a maximum gap length of 2.6 kb, indicating high continuity of the chromosome‐scale assembly (Table S4).

Three independent approaches further confirmed the high accuracy and completeness of the assembled genome. First, Hi‐C interaction heatmaps showed intense intra‐chromosomal interactions, readily detectable inter‐homologous interactions, and minimal background interactions between non‐homologous chromosomes, confirming the correct assignment of sequences to the four homologous sets (Figure 1d). Second, BUSCO analysis identified 1584 (98.1%), 1573 (97.5%), 1580 (97.9%), and 1563 (96.8%) of the 1614 conserved genes in the four monoploid genomes, respectively (Table S5). Third, Illumina short reads mapped back to the four haplotype‐resolved assemblies with high coverage rates of 99.06, 98.98, 99.11, and 99.16%, respectively (Table S6).

### Genome annotation

Repeat annotation revealed that repetitive elements occupy a substantial proportion of the autotetraploid *D. nipponica* genome. Transposable elements (TEs) accounted for approximately 66.89–67.38% of each monoploid genome, with long terminal repeat (LTR) retrotransposons being the dominant class, representing 53.09–53.68% of the genome (Figure 1e; Table S7). Among LTRs, the Gypsy and Copia superfamilies were the most abundant, contributing 25.64–25.98% and 4.83–4.87% of the genome, respectively (Table S7). In addition, tandem repeats comprised an additional 22.71–23.51% of the monoploid genome, including microsatellites (4.41–4.57%), minisatellites (8.70–9.01%), and satellite repeats (11.44–11.85%) (Table S8).

Gene annotation was conducted independently for each of the four monoploid haplotypes (hapA–hapD) using an integrated strategy combining *ab initio* prediction, homology‐based inference, and RNA‐seq evidence. The final high‐confidence gene sets comprised 23 684, 23 523, 23 518, and 23 372 protein‐coding genes in hapA, hapB, hapC, and hapD, respectively (Figure 1e; Table S9), indicating highly similar gene numbers across the four homologous genomes. Gene structural features were also highly conserved among haplotypes, with an average mRNA length of ~4.24–4.31 kb and an average coding sequence (CDS) length of ~1.21 kb per gene. Each gene contained approximately 5.28–5.30 exons, with mean exon lengths of ~285–287 bp and mean intron lengths of ~638–655 bp (Tables S9 and S10).

Functional annotation assigned putative functions to 97.42–97.51% of predicted protein‐coding genes in each monoploid genome based on comparisons with multiple public databases, including NR, UniProt, KEGG, GO, KOG, Pfam, and InterPro (Table S11; Figure S3). Overall, the high consistency in repeat content, gene number, structural features, and functional annotation across hapA–hapD is consistent with the conserved gene architecture expected for an autotetraploid genome, and supports the high quality and completeness of the genome annotation.

### High genome synteny and recent autotetraploidization

In agreement with the high similarity observed at the annotation level, the four haplotypes of autotetraploid *D. nipponica* exhibited extensive collinearity (Figure 2a). The numbers of syntenic genes were highly consistent across all six pairwise haplotype comparisons, ranging from 17 848 to 18 130 (hapA–hapB: 18 130; hapA–hapC: 18 125; hapA–hapD: 18 073; hapB–hapC: 18 057; hapB–hapD: 18 030; hapC–hapD: 17 848). Strong collinearity was also observed between the tetraploid haplotypes and diploid *D. nipponica*, with 17 792 syntenic genes identified between hapA and its diploid counterpart. In contrast, although overall synteny remained high between hapA and the closely related species *D. zingiberensis*, several chromosome rearrangements were detected (Figure 2a), indicating that structural variation may have contributed to species divergence within *Dioscorea* sect. *Stenophora*. Consistent with the extensive synteny among haplotypes, sequence similarity among homologous chromosomes was also remarkably high. Pairwise average nucleotide identity (ANI) among homologous chromosome pairs ranged from 97.00 to 98.92%, with most chromosome pairs exceeding 97.50% identity (Table S12).

**Figure 2:**
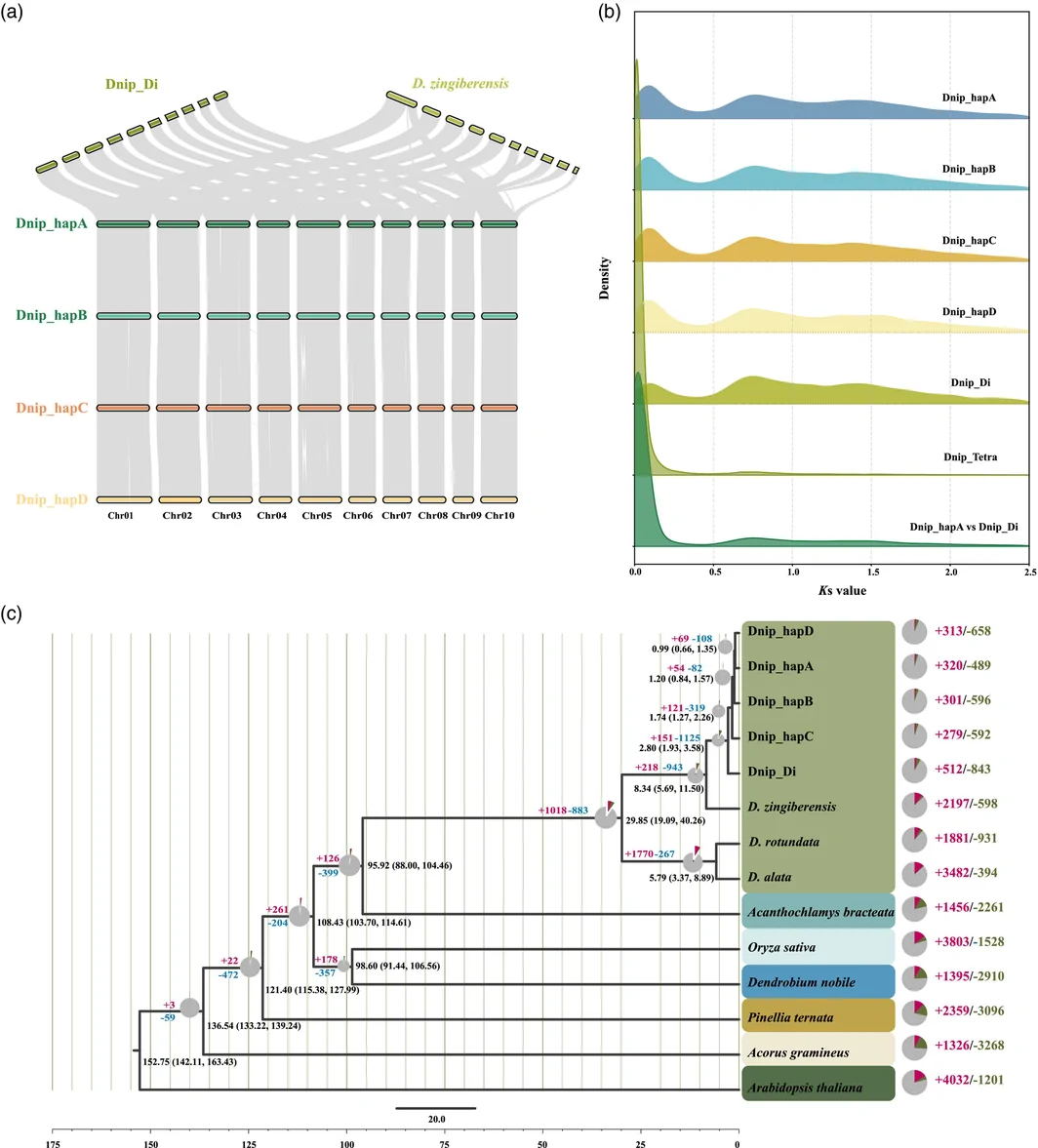
Genome evolution of autotetraploid *Dioscorea nipponica*. (a) Syntenic blocks among the four haplotypes of autotetraploid *D. nipponica* (Dnip_hapA–Dnip_hapD), as well as between the tetraploid haplotypes and diploid *D. nipponica* (Dnip_Di), and *Dioscorea zingiberensis*. (b) Distribution of synonymous substitution rates (*K*s) for syntenic gene pairs within each tetraploid haplotype, within Dnip_Di, within tetraploid *D. nipponica* (Dnip_Tetra), and between Dnip_hapA and Dnip_Di. (c) Phylogenetic relationships among diploid and tetraploid *D. nipponica* (hapA–hapD) and nine additional species, together with analyses of gene family expansion and contraction. Numbers near the nodes indicate estimated divergence times in millions of years ago (Ma). Positive (+) and negative (−) numbers denote expanded and contracted gene families in each lineage, respectively.

To elucidate the WGD history of tetraploid *D. nipponica*, we analyzed the distributions of synonymous substitution rates (*K*s) for syntenic gene pairs (Figure 2b). *K*s peaks reflect signals of both WGD event (self‐synteny) and species divergence (interspecies synteny). The *K*s profiles of the four tetraploid haplotypes were highly congruent with each other and closely resembled those of diploid *D. nipponica* and other *Dioscorea* species (Zhang et al., 2025), all of which shared two ancient WGD events. Specifically, two major peaks at *K*s ≈ 1.4 and *K*s ≈ 0.76 likely corresponded to the monocot‐wide τ‐WGD (Sun et al., 2025) and an ancient *Dioscorea*‐specific WGD event, respectively (Figure 2b). A peak at low *K*s values (*K*s ≈ 0.090) probably reflected recent tandem or segmental duplications, associated with fragmented syntenic blocks (Figure 2b). Notably, a very recent and distinct peak (*K*s ≈ 0.013) was detected in the tetraploid *D. nipponica*, corresponding to an estimated haplotype divergence time of ~1.63 million years ago (Ma), assuming a synonymous substitution rate of *r* = 4 × 10^−9^ substitutions per site per year (Hu et al., 2025). Because *K*s‐based estimates may capture both ancestral polymorphisms retained from the diploid progenitor population and sequence divergences accumulated after genome duplication, the estimated divergence time (~1.63 Ma) should be regarded as an approximate upper temporal boundary for the origin of the autotetraploid lineage. By contrast, orthologous gene pairs between hapA of tetraploid *D. nipponica* and diploid *D. nipponica* showed *K*s values centered at ~0.023, corresponding to an estimated divergence time of ~2.88 Ma (Figure 2b). These results indicate that divergence among diploid lineages preceded the subsequent autotetraploidization event.

### Phylogenomics and genome evolution

To elucidate the evolutionary relationships and divergence times of *D. nipponica*, we analyzed its diploid and tetraploid (hapA–hapD) genomes, together with eight representative monocot species, including *D. zingiberensis*, *Dioscorea rotundata*, *Dioscorea alata*, *Acanthochlamys bracteata*, *Oryza sativa*, *Dendrobium nobile*, *Pinellia ternata*, and *Acorus gramineus*, using *Arabidopsis thaliana* as the outgroup (Figure 2c). A total of 27 272 orthogroups were identified, of which 8070 were shared across *D. nipponica* haplotypes and all sampled species (Table S13). A robust maximum likelihood (ML) phylogenetic tree was reconstructed using 288 single‐copy orthologous genes, robustly resolved the relationships among *D. nipponica* and its relatives (Figure 2c). The phylogeny confirmed a sister relationship between *D. nipponica* and *D. zingiberensis*, with an estimated divergence time of ~8.34 Ma (95% CI: 5.69–11.50 Ma). Within *D. nipponica*, diploid and tetraploid haplotypes diverged approximately 2.80 Ma (1.93–3.58 Ma), whereas the four autotetraploid haplotypes formed a well‐supported monophyletic clade with an estimated coalescence age of ~1.74 Ma (1.27–2.26 Ma) (Figure 2c). Both estimates were largely consistent with *K*s‐based analyses and indicated that intraspecific diploid divergence preceded polyploidization.

Gene family expansion/contraction analyses revealed 151 expanded families in the tetraploid *D. nipponica* clade (hapA–hapD) (Figure 2c). These genes were significantly enriched for functions associated with environmental adaptation, including responses to stimulus (GO:0043207), stress (GO:0006950), and chemical factors (GO:0042221), as well as molecular functions such as oxidoreductase (GO:0016491) and acyltransferase (GO:0016746) activities (Table S14; Figure S4). KEGG pathway analysis further supported this adaptive profile, revealing significant enrichment of flavonoid, flavone/flavonol, and isoflavonoid biosynthesis pathways (Table S15; Figure S5).

In addition, expanded gene families were enriched in processes related to proteome homeostasis, including protein metabolic processes (GO:0019538), macromolecule modification (GO:0043412), and ubiquitin‐mediated protein modification and degradation activities (e.g., GO:0004842, GO:0019787), along with cytosolic (GO:0005829), vacuolar (GO:0005773), and membrane‐associated cellular components (Table S14; Figure S4). Consistently, KEGG pathways involved in ubiquitin‐mediated proteolysis, RNA exosome function, and mitochondrial biogenesis were also enriched, indicating coordinated regulatory processes contributing to the maintenance of transcriptome integrity and cellular homeostasis following polyploidization (Table S15; Figure S5).

### Widespread allele loss and tissue‐specific allelic expression bias

Based on the definition of alleles as homologous genes at the same locus (Liu et al., 2024; Yu et al., 2023), we identified 26 190 genes in the autotetraploid *D. nipponica* genome, comprising 87 089 alleles, with an average of 3.33 alleles per gene (Figure 3a; Table S16). Of these, 17 819 genes (68.03%) retained four alleles, while 8371 genes (31.97%) exhibited allele loss. Specifically, 2733 genes (10.43%) possessed 3 alleles, 1976 genes (7.54%) contained 2 alleles, and 3662 genes (13.98%) had a single allele (Figure 3a; Table S16).

**Figure 3:**
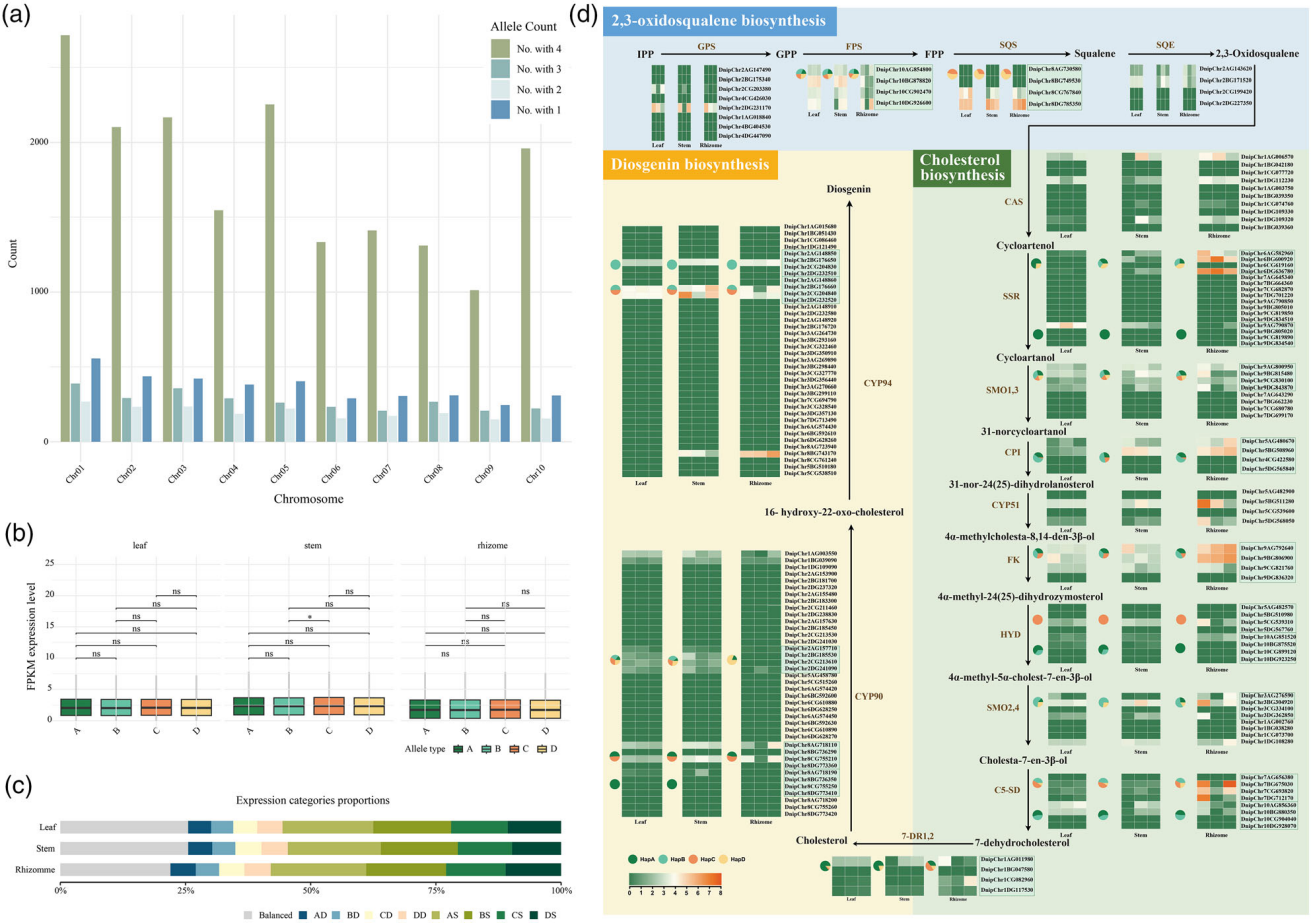
Tissue‐specific allelic expression bias in autotetraploid *Dioscorea nipponica*. (a) Distribution of genes retaining one to four alleles in the autotetraploid genome. (b) Global expression levels (FPKM) of homologous alleles from the four haplotypes (hapA–hapD) across leaf, stem, and rhizome tissues. Minor but significant differences were detected in stem tissue (*P* < 0.05). (c) Proportions of nine allelic expression categories within tetrads (sets of four homologous alleles per gene) across leaf, stem, and rhizome tissues, including balanced expression, allele‐dominant patterns (AD, BD, CD, or DD), and allele‐suppressed patterns (AS, BS, CS, or DS). (d) Allele‐specific expression patterns of genes involved in the diosgenin biosynthetic pathway in autotetraploid *D. nipponica*. The color scale represents log_2_‐transformed FPKM values [log_2_ (FPKM +1)].

Expression analysis revealed that global allele expression levels, measured as per kilobase of transcript per million mapped reads (FPKM), were highly similar across the four haplotypes (hapA–hapD) in leaf, stem, and rhizome tissues, with only minor significant differences detected in stems (*P* < 0.05) (Figure 3b). Within each tetrad (the four homologous alleles per gene), ~22–26% exhibited balanced expression, characterized by comparable transcript levels across all four homologous alleles. In contrast, the majority (55–60%) showed allele suppression (A‐suppression, AS; B‐suppression, BS; C‐suppression, CS; or D‐suppression, DS) (Figure 3c), with the highest proportion occurring in rhizome tissue.

A detailed analysis of allelic retention and expression patterns of genes involved in the diosgenin biosynthetic pathway revealed extensive allelic loss and transcriptional silencing, resulting in widespread expression bias across tissues (Figure 3d; Table S17). Among expressed tetrads, FPS and SMO1,3 tetrads exhibited balanced expression across all tissues, whereas CPI and FK were consistently D‐suppressed. In addition, one HYD tetrad and one CYP94 tetrad showed stable C‐dominant and B‐dominant expression, respectively. By contrast, pronounced tissue‐dependent regulation characterized several other pathway genes. SQS displayed D‐dominant expression specifically in stems but was A‐suppressed in leaves and rhizomes, while SMO2,4 was B‐dominant in stems and C‐suppressed in leaves and rhizomes (Figure 3d; Table S18). Notably, divergent regulation between paralogous tetrads was common. For example, one SSR tetrad was constitutively A‐dominant, whereas the other was A‐dominant in leaves but C‐suppressed in stems and rhizomes. Similarly, two CYP90 tetrads showed coordinated suppression (B‐suppressed in leaves and rhizomes and D‐suppressed in stems), whereas a third CYP90 tetrad exhibited A‐dominant expression in aerial tissues (Figure 3d; Table S18). Overall, these findings indicate that selective allelic regulation within tetrads, rather than uniform gene dosage, predominantly controls diosgenin biosynthesis in autotetraploid *D. nipponica*.

### Polyploidization reshapes rhizome metabolite profiles and gene expression in *D. nipponica*

Untargeted metabolomic analysis of rhizomes from diploid and tetraploid *D. nipponica* identified 1303 metabolites, comprising 851 in positive and 452 in negative ionization modes. These metabolites were mainly classified as lipids and lipid‐like molecules (295, e.g., fatty acids and steroids), phenylpropanoids and polyketides (181, e.g., flavonoids), organic acids and derivatives (142, e.g., amino acids and peptides), and organic oxygen compounds (101, e.g., carbohydrates) (Table S19). Among these, 60 metabolites exhibited significant differential abundance between diploid and tetraploid *D. nipponica* (Figure 4a; Table S20). Tetraploid rhizomes showed a general enrichment of flavonoids, including linarin, hesperidin, isoliquiritin, naringin dihydrochalcone, eriodictyol *O*‐malonylhexoside, and mosloflavone. In contrast, diosgenin‐type steroidal saponins, including diosgenin, diosgenin glucoside, dioscin, pseudoprotodioscin, polyphyllins, and protogracillin, were consistently reduced in tetraploids (Figure 4a; Table S20). Notably, these two metabolite classes displayed a strong negative correlation (Figure 4b), suggesting that polyploidization is accompanied by a coordinated metabolic reallocation between steroidal saponins and flavonoid pathways.

**Figure 4:**
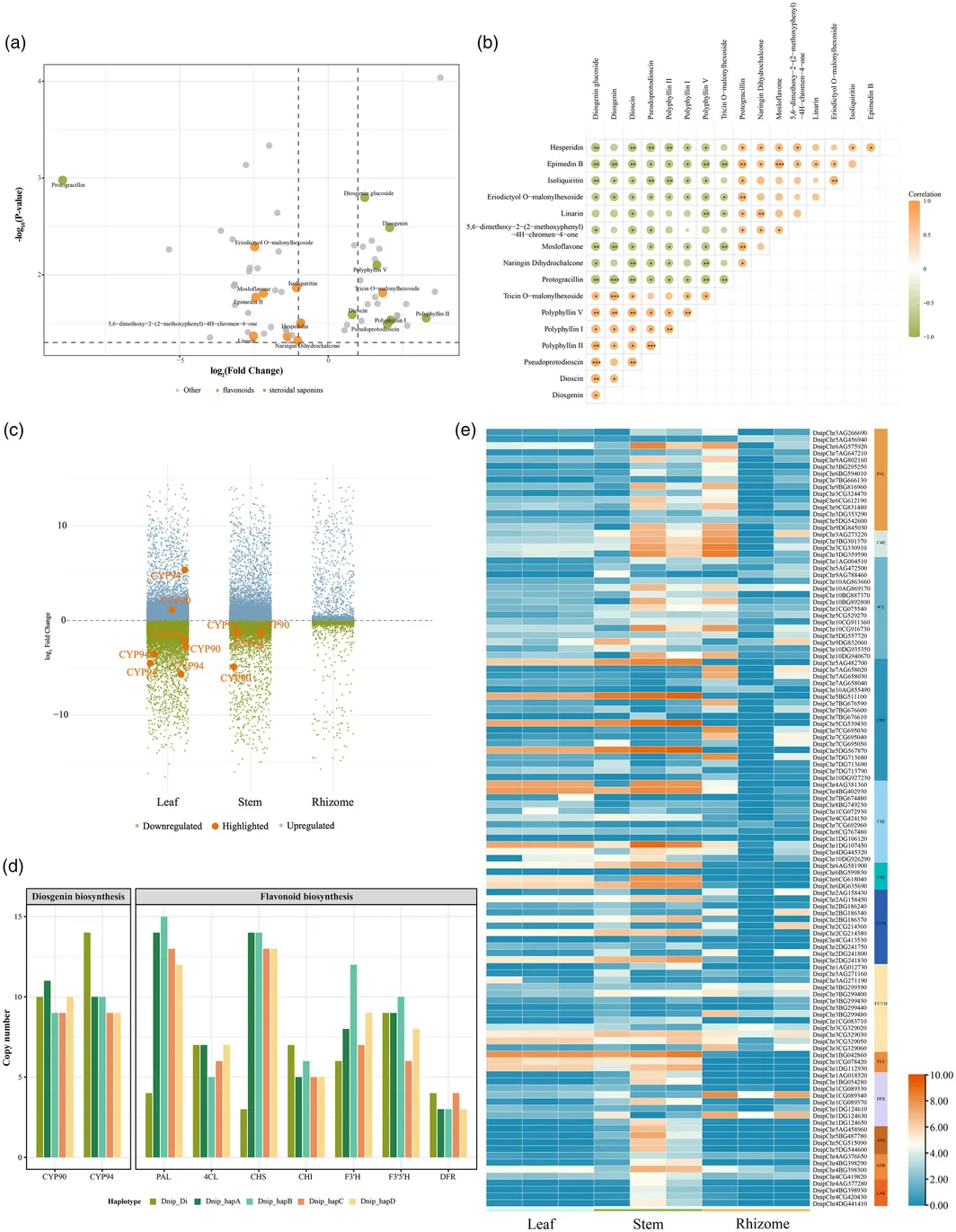
Polyploidization‐associated metabolic reprogramming and dosage‐dependent regulation of genes involved in diosgenin and flavonoid biosynthesis in *Dioscorea nipponica*. (a) Differentially accumulated metabolites in rhizomes of diploid and autotetraploid *D. nipponica*. Steroidal saponins with a diosgenin‐type backbone and flavonoid‐related metabolites are highlighted. (b) Correlation analysis between steroidal saponins and flavonoid‐related metabolites. * *P* < 0.05; ** *P* < 0.01; *** *P* < 0.001. (c) Differentially expressed genes between diploid and autotetraploid *D. nipponica* across leaf, stem, and rhizome tissues. (d) Copy number variation of key biosynthetic gene families involved in diosgenin and flavonoid pathways in diploid *D. nipponica* (Dnip_Di) and across the four haplotypes of the autotetraploid genome (Dnip_hapA–hapD). (e) Expression of transcriptionally active alleles of flavonoid biosynthetic genes in autotetraploid *D. nipponica*. The color scale represents log_2_‐transformed FPKM values [log_2_ (FPKM +1)].

At the transcriptome level, 3296, 1815, and 381 genes were significantly upregulated in leaves, stems, and rhizomes, respectively, representing positive dosage effect genes (Figure 4c; Tables S21–S23). In contrast, 4205, 1572, and 155 genes were significantly downregulated in the corresponding tissues, designated as negative dosage effect genes (Figure 4c; Tables S24–S26). GO enrichment analysis revealed a tissue‐specific transcriptional rewiring. In leaves, upregulated genes were mainly associated with fundamental cellular architecture, metabolism, and biosynthesis (Table S27), whereas downregulated genes were enriched for regulatory and stimulus‐responsive functions (Table S28). In stems, upregulated genes were mainly linked to environmental response and signaling (Table S29), while downregulated genes were enriched in core catalytic activities (Table S30). In rhizomes, upregulated genes were enriched in stress‐responsive and organelle‐related functions (Table S31), while downregulated genes were linked to core mitochondrial and metabolic processes (Table S32). Collectively, these findings highlight a tissue‐specific redistribution of metabolic, catalytic, and stress‐responsive functions, reflecting pronounced transcriptional rewiring driven by polyploidization in autotetraploid *D. nipponica*.

### Differential copy number and dosage‐dependent expression of diosgenin and flavonoid biosynthetic genes in diploid and tetraploid *D. nipponica*

To investigate the molecular basis underlying the contrasting accumulation of diosgenin and flavonoids in tetraploid *D. nipponica*, we first compared copy number variation in key biosynthetic gene families between the diploid genome and the four haplotypes of the autotetraploid genome (hapA–hapD) (Figure 4d). In the diosgenin biosynthetic pathway, CYP90 and CYP94 genes were largely stable, with the diploid genome containing 10 CYP90 and 14 CYP94 genes, and tetraploid haplotypes carrying 9–11 and 9–10 copies, respectively (Figures S6–S9). Similar copy number stability was also observed for other key enzymes in the diosgenin pathway, indicating that polyploidization had minimal impact on the copy number of diosgenin‐related genes. In contrast, gene families involved in flavonoid biosynthesis showed pronounced expansion in the tetraploid genome. For instance, the copy number of PAL increased from 4 in the diploid genome to 12–15 across tetraploid haplotypes, CHS expanded from 3 to 13–14 copies, and F3′H also showed a marked increase in gene copy number (Figure 4d; Table S33). These results indicate that polyploidization preferentially promoted the expansion of flavonoid biosynthetic gene families, which may contribute to the elevated flavonoid accumulation in tetraploid *D. nipponica*.

At the transcriptional level, a substantial proportion of alleles in diosgenin biosynthetic pathway genes exhibited low or undetectable expression levels, which may be associated with the loss of regulatory elements, such as untranslated regions (UTRs) (Figure S10). More importantly, we observed a coordinated downregulation of multiple CYP90 and CYP94 genes in leaf and stem tissues, indicating a negative dosage effect (Figure 4c; Table S34). Given their essential role in steroid backbone oxidation, this collective repression likely constitutes a major bottleneck in diosgenin biosynthesis. Additional pathway components, including SMO1, C5‐SD, and SSR, also exhibited negative dosage effects, further constraining diosgenin production (Figure 4c; Table S34). In contrast, alongside the extensive expansion of flavonoid biosynthetic genes (Figure 4d), a larger number of alleles were transcriptionally active and significant positive dosage effects were observed on several key pathway genes (e.g., C4H, 4CL, CHI, DFR, F3′5′H, FLS, ANR) (Figure 4e; Tables S35 and S36). Although negative dosage regulation was also observed in a subset of flavonoid‐related genes or gene copies (e.g., PAL, C4H, 4CL, F3′H, F3′5′H, DFR, ANS) (Figure 4e; Table S36), this repression was likely insufficient to counteract the combined influence of increased gene copy number and the coordinated upregulation of other pathway members, ultimately leading to a net increase in flavonoid accumulation.

### Population genomics reveals the origin of autotetraploid *D. nipponica*

To investigate the demographic origins of autotetraploid *D. nipponica* in southwest China, we generated a comprehensive, range‐wide population genomic dataset comprising 31 autotetraploid individuals from four populations and 91 diploid individuals from 13 populations (Figure 5a; Table S37). After stringent quality filtering, 320 839 high‐quality SNPs were retained for downstream analyses. ML phylogenetic reconstruction clearly separated autotetraploid and diploid individuals into two distinct monophyletic clades (Figure 5b). The diploid clade was further subdivided into five well‐supported subclades corresponding to their predominant geographic distributions: Southeast China (SEC), Central China (CC), Northwest China (NWC), North China (NC), and Northeast China (NEC), consistent with our previous finding (Hu et al., 2025). ADMIXTURE analysis identified six as the best‐supported number of ancestral populations (*K* = 6) (Figure S11), recovering one tetraploid group and five diploid groups, in agreement with the phylogenetic topology (Figure 5c). Principal component analysis (PCA) further corroborated this population structure (Figure 5d). To resolve finer‐scale structure within the autotetraploids, we performed additional ADMIXTURE and PCA analyses restricted to tetraploid individuals. ADMIXTURE supported *K* = 1 as the optimal model, indicating overall genetic homogeneity among autotetraploids. Nevertheless, at *K* = 2, autotetraploids separated into an eastern group (HuBYC and SaXSL) and a western group (SCBZ and CQCK) (Figure 5e), a pattern further supported by PCA (Figure 5f), suggesting subtle east–west genetic differentiation.

**Figure 5:**
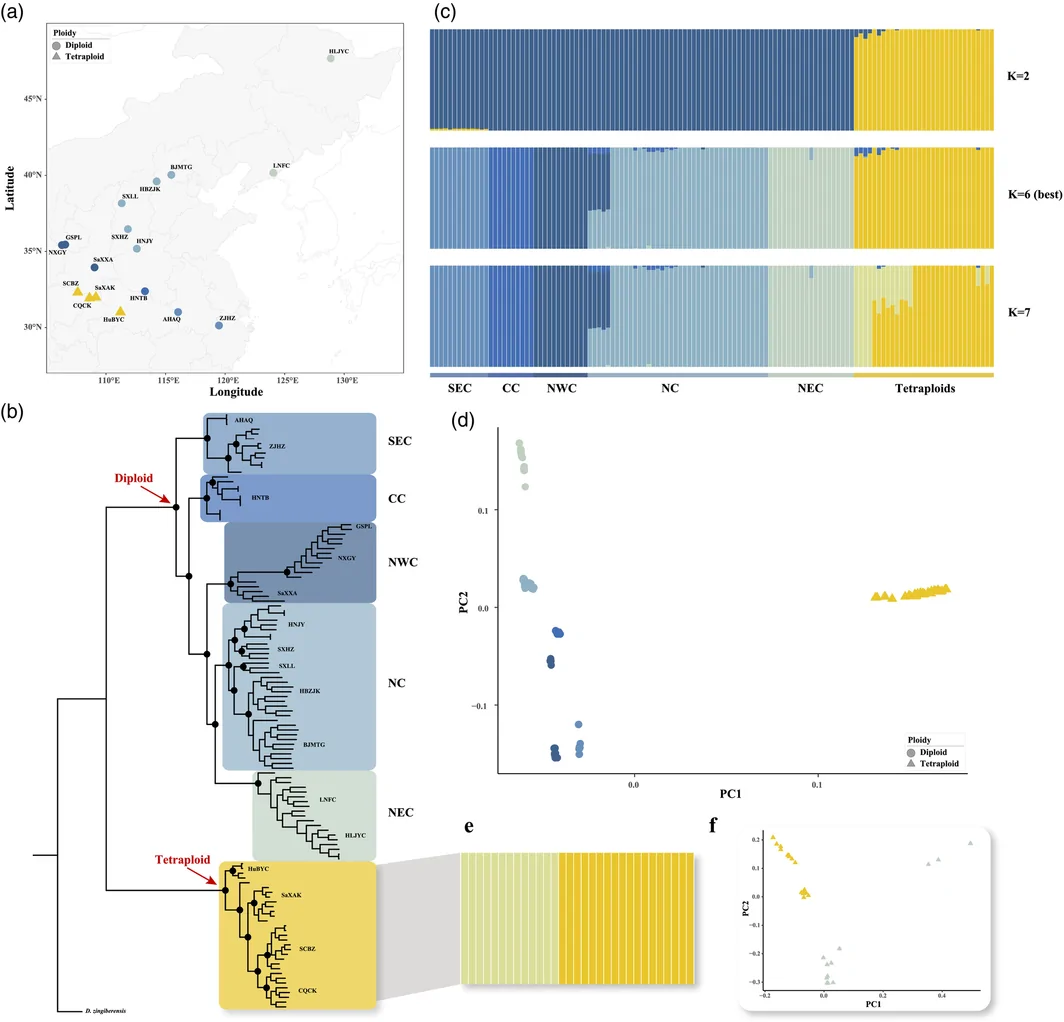
Population structure analyses of diploid and autotetraploid *Dioscorea nipponica*. (a) Geographic distribution of sampled diploid and autotetraploid *D. nipponica* populations across China. Sampling sites are colored according to ADMIXTURE results at the optimal *K* value, with triangles indicating autotetraploid populations. (b) Maximum likelihood phylogenetic tree inferred from genome‐wide SNPs of diploid and autotetraploid individuals. (c) ADMIXTURE analysis of all sampled individuals at *K* = 2, *K* = 6 (best‐supported), and *K* = 7. (d) Principal component analysis (PCA) of diploid and autotetraploid individuals. (e) ADMIXTURE analysis of autotetraploid individuals only at *K* = 2. (f) PCA of autotetraploid individuals based on genome‐wide SNP data.

Using fastsimcoal2, we inferred population divergence and demographic history of diploid and autotetraploid *D. nipponica* based on genome‐wide SNP data. The diploid populations exhibited a relatively large effective population size (*N*e), with a point estimate of 4.04 × 10^6^ (95% CI: 2.25 × 10^6^–4.22 × 10^6^). In contrast, the autotetraploid populations showed substantially reduced *N*e, estimated at 2.20 × 10^5^ (1.26 × 10^5^–1.62 × 10^6^) for the eastern tetraploid group and 1.13 × 10^5^ (3.38 × 10^4^–4.83 × 10^5^) for the western group. The divergence between the diploid ancestor of autotetraploid *D. nipponica* (with autotetraploids modeled as diploids in this analysis) and its extant diploid relative was dated to ~2.51 Ma (2.16–3.57 Ma) (Figure 6a; Table S38). Within the autotetraploid lineage, the eastern and western groups diverged more recently, at approximately 0.12 Ma (0.05–0.17 Ma), indicating subsequent regional differentiation following tetraploid formation. Relatively high and approximately symmetric recent gene flow was inferred between these two autotetraploid groups (*m*_1_ = 5.52 × 10^−5^, 5.61 × 10^−7^–8.77 × 10^−4^), initiated around 0.01 Ma (3032–49 943 years ago), suggesting secondary contact or renewed connectivity between geographically structured autotetraploid populations during the late Pleistocene. In contrast, historical gene flow between diploid populations and the ancestral autotetraploid lineage was markedly lower (*m*_2_ = 1.99 × 10^−7^, 6.40 × 10^−8^–6.28 × 10^−7^; *m*_3_ = 6.33 × 10^−8^, 1.50 × 10^−8^–3.97 × 10^−7^) (Figure 6a; Table S38).

**Figure 6:**
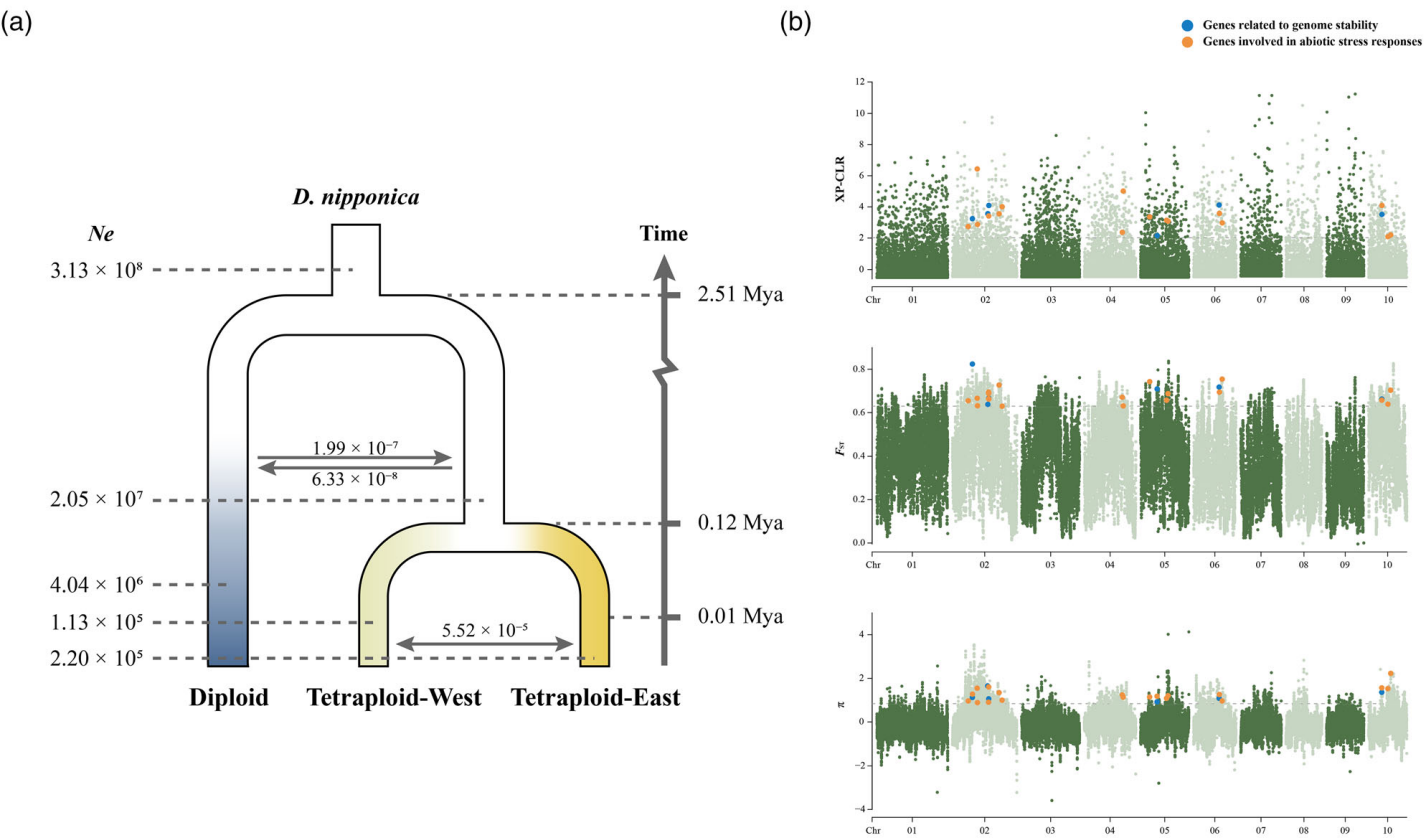
Demographic history and genomic signatures associated with autotetraploid formation and adaptation in *Dioscorea nipponica*. (a) Demographic model inferred using fastsimcoal2 based on genome‐wide SNP data from diploid and autotetraploid *D. nipponica*. The model depicts effective population sizes (*N*e), divergence times (*T*), and migration rates (*m*) among diploid populations, eastern autotetraploid populations, and western autotetraploid populations. Parameter estimates are summarized in Table S38. (b) Genome‐wide selective sweep analyses comparing autotetraploid and diploid *D. nipponica* populations. Tracks display XP‐CLR scores, *F*_ST_ values, and nucleotide diversity (*π*) ratios across chromosomes. Genes associated with genome stability and abiotic stress responses are highlighted.

### Genetic basis underlying autotetraploid establishment and adaptation

To identify candidate genes associated with autotetraploid establishment and adaptation, selective sweep analyses were conducted by comparing tetraploid populations to diploid reference populations. Genome‐wide cross‐population composite likelihood ratio (XP‐CLR), genetic differentiation (*F*_ST_), and nucleotide diversity (*π*) ratio analyses identified 2355, 2412, and 2411 candidate sweep regions, encompassing 7973, 2764, and 1466 genes, respectively (Figure 6b). The intersection of these analyses yielded 155 high‐confidence candidate genes under selection (Table S39). These included at least six genes that maintain genome stability through diverse mechanisms, such as DNA repair, recombination control, chromatin remodeling, and spindle checkpoint regulation (e.g., *AT1G19025*/Dniph105g01546, *AtTOP3*α/Dniph102g01540, *AtDMC1*/Dniph102g01574, *MCM9*/Dniph102g01253, *BUBR1*/Dniph110g01569, *CLSY2*/Dniph106g00809). Additionally, numerous genes were associated with abiotic stresses, including responses to drought (e.g., *CML37*/Dniph102g01383), salt (e.g., *MYB15*/Dniph106g00961), cold (e.g., *ANN7*/Dniph102g01564), and UV‐B radiation (e.g., *RUS2*/Dniph106g00960), as well as genes involved in biotic stress responses, including pathogen defense (e.g., *PAD3*/Dniph102g01256, *NRG1.1*/Dniph105g01981, *LYK5*/Dniph110g01571) and herbivory resistance (e.g., *AAS*/Dniph110g01967, *LAC6*/Dniph110g02053) (Figure 6b; Table S39). Several genes were also associated with developmental processes, such as flowering, meristem growth, and auxin transport (e.g., *SOC1*/Dniph102g01570, *ULT1*/Dniph105g01016, *PILS3*/Dniph110g01563) (Table S39).

Notably, a ~1 Mb interval on chromosome 2 (Chr02:35–36 Mb) harbored a dense cluster of such genes, including *AtTOP3*α (Dniph102g01540) and *AtDMC1* (Dniph102g01574), which are critical for genome stability and meiotic recombination, as well as stress‐responsive genes such as *ANN7* (Dniph102g01564), *APS1* (Dniph102g01568), and the developmental regulator *AGL20*/*SOC1* (Dniph102g01570), involved in environmental response and flowering (Figure 6b; Table S39). This region thus represents a hotspot where selection converged on genome stability, stress adaptation, and developmental regulation.

## DISCUSSION

### Chromosome‐scale assembly and expression stability of autotetraploid *D. nipponica*

Polyploidization is a prevalent and influential evolutionary force in the genus *Dioscorea*, driving speciation, genomic diversification, and phenotypic innovation (Shan et al., 2026; Sugihara et al., 2021). Despite its evolutionary and functional significance, most high‐quality reference genomes in this genus are derived from diploid species (Cheng et al., 2021; Li et al., 2022; Wang et al., 2026; Zhang et al., 2025), leaving polyploid genomes largely underrepresented. To date, only a single tetraploid genome, *D. polystachya* from section *Enantiophyllum*, has been reported (Shan et al., 2026), whereas no polyploid genome has yet been assembled for the rhizomatous section *Stenophora*. Notably, *Stenophora* represents the basal lineage sister to all remaining *Dioscorea* species (Noda et al., 2020), making it particularly informative for understanding genome evolution and the consequences of autopolyploidization within the genus. Here, we present the first chromosome‐scale genome assembly for a polyploid within *Dioscorea* sect. *Stenophora*, the tetraploid *D. nipponica*. Integrated analyses of *k*‐mer spectra, GenomeScope, Smudgeplot, and nQuire congruently supported an autotetraploid genome, which was assembled into four distinct but highly similar haplotypes (hapA–hapD) (Figure 1).

The assembly exhibited high quality and completeness, with BUSCO completeness scores ranging from 96.8 to 98.1% and low missing BUSCOs (1.0–2.0%) across the four haplotypes (Table S5). These values were comparable to, or higher than, those reported for recently published haplotype‐resolved autotetraploid plant genomes, including autotetraploid *Anoectochilus roxburghii* (~96.0%; Fang et al., 2025), *Camellia oleifera* (~95.0%; Zhang, Shi, et al., 2024), *C. paliurus* (96.8%; Yu et al., 2023), *R. officinale* (94.3–96.5%; Zhang, He, et al., 2024), and *Rhododendron nivale* subsp. *boreale* (98.8%; Lyu et al., 2024). In addition, multiple independent lines of evidence supported the robustness of the assembly, including strong Hi‐C interaction consistency, clear collinearity among homologous chromosomes, and high Illumina short‐read mapping rates (>98.98%) (Figure 1d,e; Table S6).

Consistent with a recent autopolyploid origin, the four haplotypes of autotetraploid *D. nipponica* exhibited remarkable genomic similarity in repeat composition, gene content, structural features, functional annotation, average nucleotide identity, and extensive synteny with each other and with diploid *D. nipponica* (Figures 1d,e and 2a; Tables S7–S12). At the expression level, autotetraploid *D. nipponica* also showed broadly balanced genome‐wide haplotype expression patterns, with alleles A, B, C, and D displaying comparable global FPKM distributions across tissues (Figure 3b). This equilibrium appeared to be actively regulated through a combination of genomic, transcriptional, and potentially epigenetic mechanisms. First, approximately 32% of genes retained fewer than four detectable alleles (Figure 3a), indicating substantial post‐polyploidization gene loss or fractionation. This pattern was consistent with observations in other autotetraploid genomes (e.g., Lyu et al., 2024; Yu et al., 2023; Zhang, Shi, et al., 2024), suggesting that partial allelic loss may represent a common feature of autotetraploid genome evolution and contribute to early genome stabilization and dosage balance (Gaynor et al., 2020). Second, a substantial proportion of alleles displayed low or undetectable expression levels, which might be associated with the loss of regulatory elements, such as UTRs (e.g., diosgenin biosynthesis genes; Figure S10). Third, among expression tetrads, suppressed alleles accounted for a substantial proportion (~60%) and remained stable across tissues (Figure 3c), indicating a transcriptional mechanism for genome‐wide dosage buffering. In addition, epigenetic regulation, such as DNA methylation, has been reported to play a role in stabilizing expression in autotetraploids (Zhang, Shi, et al., 2024), although its specific contribution in *D. nipponica* remained to be validated. Overall, these findings provide important insights into how autotetraploids accommodate increased gene dosage and maintain regulatory balance.

### Origin and adaptive evolution of autotetraploid *D. nipponica*

Although previous studies have suggested multiple, independent origins of polyploidy in *D. nipponica* (Hu et al., 2025), our integrated population genomic analyses supported a predominantly single origin for the sampled autotetraploid populations in southwestern China. In ADMIXTURE, PCA, and ML phylogenetic analyses, all autotetraploid individuals consistently formed a distinct group (Figure 5b–d), with analyses restricted to tetraploids revealing overall genetic homogeneity (best *K* = 1 in ADMIXTURE). *K*s‐based analysis detected a recent divergence peak among homologous haplotypes in autotetraploid *D. nipponica*, corresponding to an estimated haplotype divergence time of ~1.63 Ma (Figure 2b). However, this estimate should be interpreted cautiously because sequence divergence among homologous haplotypes may reflect both ancestral polymorphisms inherited from the diploid progenitor population and mutations accumulated after genome duplication. Therefore, the *K*s‐based estimate likely represents an upper temporal boundary for the emergence of the autotetraploid lineage rather than the precise timing of autotetraploidization. Consistently, phylogenetic analysis recovered the four autotetraploid haplotypes as a strongly supported monophyletic clade, with an estimated coalescence age of ~1.74 Ma (1.27–2.26 Ma) (Figure 2c). Together, these independent estimates indicate that the autotetraploid lineage of *D. nipponica* originated relatively recently. Importantly, both estimates postdated the divergence between the diploid ancestor of autotetraploid *D. nipponica* and its diploid relative, inferred from *K*s‐based (~2.88 Ma; Figure 2b), MCMCtree (2.80 Ma; Figure 2c) and coalescent‐based (2.51 Ma; Figure 6a) estimates, collectively confirming that intraspecific diploid divergence preceded polyploidization. Notably, this temporal window overlapped with the major climatic transition from the Pliocene to the Pleistocene (Lawrence et al., 2010; Lisiecki & Raymo, 2007). During this period, intensified tectonic uplift and associated geomorphological changes likely caused pronounced landscape fragmentation, isolating populations into distinct habitats and facilitating genetic divergence (Zhang et al., 2011). Concurrently, shifts toward colder and drier climatic conditions may have imposed strong environmental stresses that favored WGD (Feng et al., 2024; Li et al., 2019).

The genomic signatures of autotetraploid *D. nipponica* provided insight into its potential adaptations to these climatic challenges. Strong selective signals were detected in genes associated with genome stability, stress response, and developmental regulation (Figure 6b; Table S39). Key meiotic and DNA repair genes, such as *AtTOP3α*, which suppresses somatic crossovers and resolves meiotic recombination intermediates (Hartung et al., 2008), and *AtDMC1*, responsible for repairing DNA double‐strand breaks (Pradillo et al., 2014), were under selection, likely ensuring faithful chromosome pairing and segregation in the newly duplicated genome. Simultaneously, genes mediating abiotic stress responses, including the drought‐responsive *CML37* (Scholz et al., 2015) and cold‐responsive *ANN7* (Ghosh et al., 2021), were also targeted, potentially facilitating adaptation to the colder, drier Pleistocene climate. Thus, selection on these genes likely enhanced the adaptive capacity of autotetraploid *D. nipponica*, enabling it to thrive in marginal habitats and maintain ecological success under challenging environmental conditions.

### Metabolic rewiring in autotetraploid *D. nipponica*: enhanced flavonoid accumulation at the expense of diosgenin biosynthesis

Our metabolomic analysis provided strong evidence that WGD can induce differential accumulation of secondary metabolites across biosynthetic pathways, increasing the levels of some compounds while decreasing others (Gaynor et al., 2020). In autotetraploid *D. nipponica*, rhizome metabolite profiles shifted markedly: flavonoids were enriched, whereas diosgenin‐type steroidal saponins were consistently reduced relative to the diploid (Figure 4a,b; Table S20). These patterns indicated a metabolic trade‐off, likely reflecting competition between flavonoid and MEP/diosgenin pathways for shared carbon resources (Xie et al., 2008), with carbon flux preferentially channeled toward flavonoid biosynthesis in the autotetraploid.

Mechanistically, this shift appeared to be driven by both gene copy number variation and transcriptional regulation. Diosgenin biosynthetic gene families (CYP90, CYP94) remained largely stable across ploidy levels, whereas key flavonoid pathway genes (e.g., PAL, CHS, F3′H) underwent notable expansion in the tetraploid genome (Figure 4d; Table S33), a finding also supported by KEGG enrichment analysis (Table S15). Transcriptomic analysis further revealed negative dosage effects in the diosgenin pathway, with multiple genes (e.g., CYP90, CYP94, SMO1, C5‐SD) downregulated in tetraploid vegetative tissues, likely creating bottlenecks in steroid backbone oxidation (Figure 4c; Table S34). In contrast, several flavonoid genes (e.g., C4H, 4CL, DFR) exhibited strong positive dosage effects consistent with increased pathway output (Table S36). Although a subset of flavonoid genes were negatively regulated (Table S36), the overall outcome was an increase in flavonoid production (Figure 4a,b; Table S20).

This metabolic shift toward flavonoid accumulation may enhance the ecological resilience of autotetraploid *D. nipponica*. Flavonoids contribute to abiotic stress tolerance by mitigating oxidative damage under drought, cold, and other environmental stresses (Dong & Lin, 2021; Jiang et al., 2016; Nakabayashi & Saito, 2015) and also function as floral pigments, potentially improving pollinator attraction and reproductive assurance (Forkmann, 1991; Grotewold, 2006; Rudall, 2020). Consistent with this adaptive potential, polyploid *D. nipponica* frequently occupies range margins, with autotetraploids in southwest China and Japan, hexaploids in northeastern China, and octoploids in southwest China (Hu et al., 2025). Together, this distribution pattern and the metabolic shift toward flavonoid accumulation suggest that WGD may have contributed to enhanced stress tolerance and local persistence in peripheral or suboptimal habitats, promoting population establishment and episodic expansion.

## EXPERIMENTAL PROCEDURES

### Plant materials, genome, and transcriptome sequencing

Fresh leaves for genomic DNA extraction were collected from a single tetraploid *D. nipponica* individual originating from a wild population in Chengkou County (108°39′46″ E, 31°46′32″ N), Chongqing, China. The plant had been *ex situ* conserved for 3 years in the Yam (*Dioscorea*) Germplasm Resource Nursery of Institute of Botany, Jiangsu Province and Chinese Academy of Sciences prior to leaf sampling. High‐quality DNA was extracted from fresh leaves by a modified CTAB protocol and quantified using NanoDrop One (Thermo Fisher Scientific) and Qubit 3.0 (Life Technologies, Carlsbad, CA, USA).

A short‐read library was constructed using the Nextera DNA Flex Library Prep Kit (Illumina, San Diego, CA, USA) with random fragmentation and PCR amplification, targeting insert sizes of 200–400 bp. Paired‐end sequencing (PE 150 bp) was performed on the Illumina NovaSeq 6000 platform, generating 92.39 Gb of raw data (41.62× coverage, based on a genome size estimated by flow cytometry) for the initial genome survey (Table S40). For long‐read sequencing, a PCR‐free SMRTbell library was constructed using the SMRTbell Express Template Prep Kit 2.0 (PacBio, Menlo Park, CA, USA) and sequenced on the PacBio Sequel II platform, yielding approximately 84.67 Gb of high‐fidelity (HiFi) reads with an average genome coverage of 38.14× (Table S1). Additionally, a Hi‐C library was constructed following the standard protocol (Lieberman‐Aiden et al., 2009) and sequenced on the Illumina NovaSeq 6000 platform (PE 150 bp), producing 257.00 Gb of data (115.77× coverage) for chromosome‐scale genome scaffolding (Table S3). To support genome annotation, leaves, stems, and rhizomes were collected from the same individual. Total RNA extraction, library construction, and sequencing followed the protocol used for diploid transcriptome sequencing in our previous study (Hu et al., 2025).

For gene expression analyses, including allele‐specific expression bias in autotetraploids and expression dosage effects between diploids and autotetraploids, total RNA was extracted from leaves, stems, and rhizomes of both diploid and tetraploid individuals, with three biological replicates per tissue and ploidy. RNA sequencing was performed on the Illumina NovaSeq 6000 platform using paired‐end 150 bp reads, yielding approximately 6 Gb of raw data per sample.

### Genome survey, assembly and assessment

Raw reads were filtered with fastp v0.23.2 (Chen et al., 2018) to remove adaptor sequences and low‐quality reads. The resulting clean reads were subjected to *k*‐mer analysis (*k* = 19) using KMC v3.2.4 (Kokot et al., 2017). Genome size was estimated as the total number of *k*‐mers divided by the depth of the major peak, while genome heterozygosity and repeat content were inferred using GenomeScope 2.0 (Ranallo‐Benavidez et al., 2020). Genome ploidy and structure were further evaluated with Smudgeplot v0.4.0 (Ranallo‐Benavidez et al., 2020), by comparing the sum of *k*‐mer pair coverages (CovA + CovB) to their relative coverage (CovB/(CovA + CovB)). Additionally, genome ploidy was inferred using nQuire (Weiß et al., 2018) based on base frequency distributions at variable sites.

The genome of autotetraploid *D. nipponica* was assembled from PacBio HiFi reads using hifiasm v0.25.0 (Cheng et al., 2022) with Hi‐C integration (‐‐h1, ‐‐h2), generating a primary assembly and two phased contig‐level assemblies (hap1 and hap2). The two phased haplotypes were merged and subjected to chromosome‐scale phasing using CPhasing v0.2.1 (https://github.com/wangyibin/CPhasing) with the parameter ‐n 10:4, which first clustered contigs into 10 homologous groups (corresponding to the basic chromosome number *x* = 10) and then resolved each group into four haplotypes, yielding 40 pseudochromosomes. The CPhasing output was subsequently processed using Juicer v1.6 (Durand et al., 2016) and 3D‐DNA v180419 (Dudchenko et al., 2017) to generate Hi‐C contact maps (.hic) and assembly files for downstream visualization and manual curation in Juicebox v2.20 (Robinson et al., 2018). Contig order and orientation were manually corrected based on Hi‐C interaction patterns, and redundant contigs lacking Hi‐C support were removed. The final chromosome‐level assembly was obtained by linking ordered contigs with gap‐filling Ns, while contigs without sufficient interaction signals were retained as unanchored sequences. Genome assembly completeness was evaluated using BUSCO v5.4.7 based on 1614 conserved orthologous genes with default parameters (Manni et al., 2021). Additionally, Illumina short reads were mapped to the assembled genome using BWA v0.7.17 to assess coverage and sequencing depth (Li, 2013).

### Genome annotation

Repetitive sequences were classified into interspersed repeats (TEs) and tandem repeats. Interspersed repeats were identified using both *de novo* and homology‐based approaches. For *de novo* identification, RepeatModeler v2.0.11 (Flynn et al., 2020), LTR_FINDER v1.07 (Xu & Wang, 2007), LTRharvest v1.6.5 (Ellinghaus et al., 2008), and LTR_retriever v3.0.1 (Ou & Jiang, 2018) were used to construct *de novo* repeat libraries, and RepeatMasker v4.1.7 (Chen, 2004) was applied to annotate genome‐wide repeats based on the *de novo* library. For homology‐based searches, RepeatMasker was used with the RepBase database (https://www.girinst.org/repbase) to identify known repeat elements. Tandem repeats were detected using TRF v4.09 (Benson, 1999).

Gene structure annotation was performed by integrating *ab initio*, homology‐based, and transcriptome‐assisted predictions using a repeat‐masked genome. For *ab initio* prediction, AUGUSTUS v3.5.0 (Stanke et al., 2008) and GlimmerHMM v3.0.4 (Delcher et al., 2007) were applied with the default parameters. For homology‐based prediction, protein sequences from three closely related species (i.e., *D. alata*, *D. rotundata*, and *D. zingiberensis*) and diploid *D. nipponica* were aligned to the genome using MiniProt v0.13 (Li, 2023) to generate gene models. For transcriptome‐assisted prediction, RNA‐seq reads from leaves, stems, and rhizomes were aligned to the genome using HISAT2 v2.2.1 (Kim et al., 2019), transcripts were assembled with StringTie v2.2.1 (Pertea et al., 2015), and coding sequences were predicted using TransDecoder v5.7.0 (https://github.com/TransDecoder/TransDecoder). Gene models from all approaches were integrated with MAKER2 v3.01.03 (Holt & Yandell, 2011) to generate a non‐redundant gene set. Functional annotation of the predicted proteins was carried out using DIAMOND v2.1.8 (Buchfink et al., 2015) and hmmscan v3.3.2 (Eddy, 2009), with searches conducted against seven public databases, including NR (Deng et al., 2006), UniProt (Apweiler et al., 2004), KEGG (Aramaki et al., 2020), GO (Ashburner et al., 2000), KOG (Koonin et al., 2004), Pfam (Finn et al., 2014), and InterPro (Mulder & Apweiler, 2007) databases.

### Genome synteny and WGD analysis

Genome‐wide synteny between tetraploid (using haplotype A as representative) and diploid *D. nipponica*, between tetraploid *D. nipponica* and *D. zingiberensis*, and among the four haplotypes of the tetraploid genome (hapA–hapD) was identified and visualized using JCVI v1.5.11 (Tang et al., 2024). ANI between each homologous chromosome pair was calculated using FastANI v1.34 (Jain et al., 2018). To investigate WGD and lineage divergence events, protein sequences from the four tetraploid haplotypes, diploid *D. nipponica* (Dnip_Di), and tetraploid *D. nipponica* (Dnip_Tetra) were aligned using BLASTP (*E*‐value <1e^−5^) to identify both paralogous gene pairs (within genomes) and orthologous pairs (between hapA and Dnip_Di). Collinear gene pairs were then detected from these alignments using WGDI with the ‐icl parameter, and their synonymous substitution rates (*K*s) were calculated using the ‐ks option. The timing of WGD and lineage divergence events was estimated using the formula *T* = *K*s/2*r*, with *r* = 4 × 10^−9^ substitutions per site per generation (Hu et al., 2025).

### Phylogenomic and gene family analyses

Orthologous gene groups were identified among the four haplotypes of tetraploid *D. nipponica* (hapA–hapD), diploid *D. nipponica*, eight monocot species (*D. zingiberensis*, *D. rotundata*, *D. alata*, *A. bracteata*, *O. sativa*, *D. nobile*, *P. ternata*, *A. gramineus*), and the outgroup *A. thaliana*, using OrthoFinder v2.5.5 with default parameters (Emms & Kelly, 2019). Single‐copy orthologous genes were aligned with MUSCLE v5.1 (Edgar, 2004), trimmed with trimAl v1.4 (Capella‐Gutiérrez et al., 2009), and concatenated into a supergene alignment matrix for phylogenetic inference. ML phylogenetic tree was reconstructed using RAxML v8.2.12 (Stamatakis, 2014) with 1000 bootstrap replicates. Divergence times were estimated using MCMCTree in PAML v4.10.0 (Yang, 2007), applying the following calibration points from Hu et al. (2025) and the Timetree database (http://www.timetree.org): (i) *D. nipponica* and *D. zingiberensis* (6.2–20.0 Ma), (ii) *D. nipponica* and *D. alata* (16.6–48.3 Ma), and (iii) *Dioscorea* and *Acanthochlamys* (86.1–132.6 Ma). Gene family expansion and contraction were analyzed using CAFÉ (Han et al., 2013), and functional enrichment analyses of GO terms and KEGG pathways were conducted using TBtools v2.337 (Chen et al., 2023).

### Allele identification and expression bias analysis

Alleles were identified as homologous genes at the same locus across the four haplotypes of the autotetraploid *D. nipponica* genome using AlleleFinder v1.2.1 (Zhang et al., 2018), with hapA as the reference. To assess allelic expression patterns among the four haplotypes, RNA‐seq data from tetraploid leaves, stems, and rhizomes were aligned to the haplotype‐resolved genome (hapA–hapD) using HISAT2 v2.2.1 (Kim et al., 2019). To minimize potential misassignment among highly similar homologous sequences, only uniquely mapped reads were retained for FPKM quantification by FeatureCounts v2.0.1 (Liao et al., 2014). Expression bias analysis was restricted to allele tetrads with a 1:1:1:1 correspondence across the four haplotypes. A tetrad was considered an expression tetrad if the sum of FPKM values across its four constituent alleles (A, B, C, and D) exceeded a threshold of 0.5 in a given tissue, a commonly used threshold in polyploid expression studies to exclude low expression noise (Zhang, Shi, et al., 2024). For each expression tetrad, the relative expression contribution of each allele was calculated as its FPKM proportion relative to the total tetrad expression (e.g., Expression(A) = FPKM(A)/[FPKM(A) + FPKM(B) + FPKM(C) + FPKM(D)]). Expression bias was categorized by calculating the Euclidean distance between each tetrad's normalized expression profile and nine predefined idealized patterns: Balanced (0.25, 0.25, 0.25, 0.25), A/B/C/D‐dominant (e.g., A‐dominant: 1, 0, 0, 0), and A/B/C/D‐suppressed (e.g., A‐suppressed: 0, 0.33, 0.33, 0.33). Each tetrad was assigned to the category with the shortest distance. The custom scripts used for allele‐specific expression analysis and figure generation are publicly available at GitHub (https://github.com/thisis33/tetraploid‐Dioscorea‐nipponica‐analysis‐scripts).

### Metabolomic profiling and transcriptome analysis of diploid and tetraploid *D. nipponica*

Diploid seedlings from Tongbai Mountain, Henan, China, and tetraploid seedlings from Chengkou County, Chongqing, China, were co‐cultivated for 3 years in the Yam (*Dioscorea*) Germplasm Resource Nursery of the Institute of Botany, Jiangsu Province and Chinese Academy of Sciences, to minimize environmental effects. Fresh rhizome tissues were harvested, immediately frozen in liquid nitrogen, and stored at −80°C prior to metabolite extraction. Three biological replicates were collected for each ploidy level. Untargeted metabolomics analysis was conducted by Novogene Bioinformatics Technology Co. Ltd. (Beijing, China) using an ultra‐performance liquid chromatography–tandem mass spectrometry (UPLC‐MS/MS) system. Raw UPLC‐MS/MS data were processed using Compound Discoverer 3.1 (Thermo Fisher Scientific, Waltham, MA, USA) to perform peak alignment, peak picking, and metabolite quantitation. These metabolites were annotated using the KEGG database (https://www.genome.jp/kegg/pathway.html), HMDB (https://hmdb.ca/metabolites), and LIPID Maps (http://www.lipidmaps.org/) databases. PCA and partial least squares–discriminant analysis (PLS‐DA) were employed to evaluate global metabolic variation between ploidy levels. Differentially accumulated metabolites (DAMs) were identified based on the combined criteria of variable importance in projection (VIP) >1.0, fold change (FC) >1.2 or <0.833, and a *P*‐value <0.05 as determined by univariate *t*‐tests.

To further elucidate gene expression patterns underlying the functional consequences of polyploidization, RNA‐seq data from leaves, stems, and rhizomes of diploid and autotetraploid individuals were aligned to the diploid *D. nipponica* genome using HISAT2 v2.2.1 (Kim et al., 2019), followed by normalization to FPKM with FeatureCounts v2.0.1 (Liao et al., 2014). Differential expression analysis was performed with the DESeq2 package (Love et al., 2014), with thresholds set at an adjusted *P*‐value (*P*adj) <0.05 and |log_2_FC| ≥1. Genes upregulated in autotetraploids relative to diploids were classified as exhibiting an expression dosage effect, whereas genes showing higher expression in diploids were considered to display a negative expression dosage effect (Qu et al., 2023).

### Comparative identification and expression analysis of diosgenin and flavonoid biosynthetic genes between diploid and tetraploid *D. nipponica*

Genes involved in the diosgenin biosynthetic pathway in the autotetraploid *D. nipponica* genome were identified following our previous framework (Hu et al., 2025). For early‐ and intermediate‐step genes (including GPS, FPS, SQS, SQE, CAS, SSR, CPI, CYP51, FK, HYD, C5‐SD, 7‐DR, and SMO), protein sequences from *A. thaliana*, *O. sativa*, and *Zea mays* were retrieved from the KEGG database and used as queries for BLASTP searches against protein sequences of the four tetraploid haplotypes (hapA–hapD), with an *E*‐value threshold of 1 × 10^−5^, sequence identity >50%, and bit score >200. For cytochrome P450 (CYP) genes, a combined HMM‐ and BLASTP‐based identification strategy was employed. Specifically, the conserved CYP domain (PF00067) was used to search against protein sequences of tetraploid haplotypes using HMMER v3.3 (Eddy, 2011), while *A. thaliana* CYP sequences were used in parallel for BLASTP searches. Only candidates supported by both methods were retained, and proteins shorter than 300 amino acids or longer than 600 amino acids were excluded. To classify CYPs into subfamilies, multiple sequence alignments were performed with MUSCLE v5.1 (Edgar, 2004), incorporating *A. thaliana* homologs as references, followed by ML phylogenetic analysis using IQ‐TREE v2.2.2.9 with 1000 bootstrap replicates (Nguyen et al., 2015). Exon–intron structures of genes involved in the diosgenin biosynthetic pathway were analyzed and visualized using GSDS v2.0 (Hu et al., 2015).

For flavonoid biosynthesis, core gene families (PAL, CHS, and CHI) were identified in the tetraploid haplotypes and diploid genome using HMMER v3.4 with Pfam domain profiles (PF00221 for PAL, PF00195 and PF02797 for CHS, PF02431, PF16035, and PF16036 for CHI). Additional pathway genes (C4H, 4CL, F3H, F3′H, F3′5′H, FLS, DFR, ANS, ANR, and LAR) were identified by BLASTP searches using reference sequences (Liu et al., 2022), applying thresholds of *E*‐value ≤1 × 10^−5^, sequence identity >50%, and bit score >150. Gene expression levels were extracted from transcriptome datasets, and both gene copy number and expression patterns were systematically compared across haplotypes and between ploidy levels.

### Population resequencing and SNP calling

A total of 23 tetraploid *D. nipponica* individuals from three populations in southwestern China were newly collected in this study (Table S37). Genomic DNA was extracted from leaf tissue using DNA Plantzol (Invitrogen, Waltham, MA, USA). Paired‐end sequencing (150 bp) was performed on the Illumina NovaSeq 6000 platform, generating 15.44–19.84 Gb of raw data per sample. These newly generated data were combined with previously published datasets (Hu et al., 2025), resulting in a final dataset comprising 31 tetraploid individuals from four populations and 91 diploid individuals from 13 populations for population genomic analyses (Table S37).

Raw reads were first filtered using fastp v0.23.2 (Chen et al., 2018) with default parameters. Clean reads from each sample were aligned to the diploid *D. nipponica* reference genome using the ‘mem’ algorithm in BWA v0.7.12 (Li, 2013). The resulting alignments were converted to BAM format, sorted, and indexed with SAMtools v1.14 (Li et al., 2009), and PCR duplicates were marked with Picard v2.1.9 (http://broadinstitute.github.io/picard/). Variant calling for each sample was performed using GATK HaplotypeCaller (McKenna et al., 2010) in ‐ERC GVCF mode, following a two‐step strategy. First, all 122 samples were analyzed under a diploid model (‘‐‐sample‐ploidy 2’) to enable joint genotyping across ploidy levels, followed by tetraploid‐specific variant calling using ‘‐‐sample‐ploidy 4’. Individual GVCFs were merged using CombineGVCFs, followed by joint genotyping with GenotypeGVCFs. SNPs were then hard‐filtered using GATK VariantFiltration with the following criteria: QD <2.0 || MQ <40.0 || FS >60.0 || SOR >3.0 || MQRankSum <−12.5 || ReadPosRankSum <−8.0.

### Population genomic structure and demographic analyses

Population genomic structure was initially assessed using a combined dataset of diploid and tetraploid individuals, followed by separate analyses focusing exclusively on tetraploids. For tetraploid‐specific analyses, high‐quality SNPs were converted to pseudo‐diploid genotypes by randomly sampling two alleles per locus. In both analyses, SNPs were filtered using VCFtools v0.1.16 to include only biallelic sites with a minor allele frequency (MAF) ≥0.05 and ≤10% missing data, and subsequently thinned to one SNP per 1 kb window. A ML phylogenetic tree was reconstructed from genome‐wide SNPs using RAxML v8.2.12 (Stamatakis, 2014) with 100 bootstrap replicates. Population structure was inferred using ADMIXTURE v1.3.0 (Alexander et al., 2009), testing *K* values from 1 to 10 for the combined dataset and 1 to 4 for tetraploid‐only analyses; the optimal *K* was selected based on cross‐validation error. PCA was performed to examine clustering of individuals and populations using VCFtools v0.1.16 (Danecek et al., 2011) and PLINK v1.90 (Chang et al., 2015).

Based on the inferred phylogenetic relationships and population structure, a demographic model (Figure 6a) was specified to estimate key demographic parameters, including divergence time (*T*), effective population size (*N*e), and gene flow (*m*), between diploid and autotetraploid lineages, as well as between the two autotetraploid groups, using fastsimcoal2 (Excoffier et al., 2021). Two‐dimensional joint site frequency spectra (2D‐SFS) were constructed with easySFS (https://github.com/isaacovercast/easySFS). For parameter estimation, 100 000 coalescent simulations were conducted per run to generate the expected SFS, which was compared to the observed 2D‐SFS to calculate composite likelihoods. The run with the highest likelihood among 100 replicate runs was used to estimate demographic parameters. To obtain 95% confidence intervals (CIs), 100 parametric bootstrap replicates were performed by simulating SFS datasets from the ML parameter estimates.

### Detection of selective sweeps

To identify genomic regions associated with the establishment and adaptation of autotetraploid *D. nipponica*, selective sweep analyses were performed focusing on tetraploid populations, with diploid populations serving as the reference. Signals of selection were detected through a combined analysis of cross‐population composite likelihood ratio (XP‐CLR), genetic differentiation (*F*_ST_), and nucleotide diversity ratios (*π*_diploid_/*π*_tetraploid_). XP‐CLR analysis was conducted with a window size of 100 kb, a step size of 10 kb, a maximum of 200 SNPs per window, and a linkage disequilibrium cutoff of 0.95 (Chen et al., 2010). *F*_ST_ and *π* were calculated using VCFtools v0.1.16 (Danecek et al., 2011) with a sliding window of 100 kb and a step size of 10 kb. Putative selective sweeps were defined as genomic regions corresponding to the top 5% of observed values for each statistic. Genes located in sweep regions identified by all three methods were considered high‐confidence candidate genes underlying autotetraploid establishment and adaptation. Gene annotation was conducted using *A. thaliana* orthologues from TAIR (https://www.arabidopsis.org; Reiser et al., 2024).

## ACCESSION NUMBERS

The genome assembly and annotation files of tetraploid *Dioscorea nipponica* (hapA–hapD) have been deposited in Figshare (https://figshare.com/s/66604fcd8a78b418a74e). Whole‐genome sequencing data generated for genome assembly and transcriptome profiling have been deposited in the NCBI Sequence Read Archive (SRA) under BioProject accession PRJNA1414691. Illumina short‐read, PacBio HiFi, and Hi‐C sequencing data are available under SRR37115708, SRR37115707, and SRR37115706, respectively. RNA‐Seq datasets from leaf, stem, and rhizome tissues of both diploid and tetraploid *D. nipponica* are available under SRR37115700–SRR37115705 and SRR37160612–SRR37160629. In addition, whole‐genome resequencing data from natural populations have been deposited under a separate BioProject PRJNA1470285, with SRA accession numbers SRR38827304–SRR38827470.

## AUTHOR CONTRIBUTIONS

R‐SL conceived the project. RL, SL, and XS designed the experiments. PL, YL, J‐HG, and MC collected samples. R‐SL, S‐SL, YF, KH, and YL analyzed data. R‐SL, S‐SL, and X‐QS wrote the manuscript. All authors read and approved the manuscript. S‐SL and X‐QS contributed equally to this work.

## CONFLICT OF INTEREST

All authors confirm that they have no conflict of interest.

## Supporting information


**Figure S1.** Genome size estimation of autotetraploid *Dioscorea nipponica* by flow cytometry.
**Figure S2.** *K*‐mer spectra and fitted models for autotetraploid *Dioscorea nipponica*.
**Figure S3.** Functional annotation of (a) hapA, (b) hapB, (c) hapC, and (d) hapD of autotetraploid *Dioscorea nipponica* based on the NR, UniProt, KEGG, GO, KOG, Pfam, and InterPro databases.
**Figure S4.** GO enrichment analysis of expanded genes in the autotetraploid *Dioscorea nipponica*.
**Figure S5.** KEGG enrichment analysis of the top 10 expanded gene families in the autotetraploid *Dioscorea nipponica*.
**Figure S6.** Phylogenetic analysis of CYP gene families in the hapA of tetraploid *Dioscorea nipponica* and *Arabidopsis thaliana*.
**Figure S7.** Phylogenetic analysis of CYP gene families in the hapB of tetraploid *Dioscorea nipponica* and *Arabidopsis thaliana*.
**Figure S8.** Phylogenetic analysis of CYP gene families in the hapC of tetraploid *Dioscorea nipponica* and *Arabidopsis thaliana*.
**Figure S9.** Phylogenetic analysis of CYP gene families in the hapD of tetraploid *Dioscorea nipponica* and *Arabidopsis thaliana*.
**Figure S10.** Exon–intron structures of genes involved in the diosgenin biosynthetic pathway.
**Figure S11.** ADMIXTURE cross‐validation error across *K* values.

**Table S1.** HiFi sequencing statistics for autotetraploid *D. nipponica*.
**Table S2.** Statistics of the HiFi‐based preliminary genome assembly of autotetraploid *D. nipponica*.
**Table S3.** Statistics of Hi‐C sequencing data for autotetraploid *D. nipponica*.
**Table S4.** Chromosome‐scale genome assembly statistics for autotetraploid *D. nipponica*.
**Table S5.** The completeness evaluation of the tetraploid *D. nipponica* genome using the BUSCO approach.
**Table S6.** Genome assembly assessment based on Illumina short‐read mapping.
**Table S7.** Statistics of the transposable elements (TEs) in the tetraploid *D. nipponica* genome.
**Table S8.** Statistics of the tandem repeats in the tetraploid *D. nipponica* genome.
**Table S9.** Predicted protein‐coding gene models for each haplotype of tetraploid *D. nipponica*.
**Table S10.** Statistics of protein‐coding gene predictions in the haplotypes of tetraploid *D. nipponica* genome.
**Table S11.** Functional annotation of genes in the tetraploid *D. nipponica* genome.
**Table S12.** Pairwise average nucleotide identity (ANI) and alignment statistics among homologous chromosome pairs in autotetraploid *D. nipponica*.
**Table S13.** Summary statistics of OrthoFinder orthogroup inference.
**Table S14.** Gene Ontology (GO) enrichment analysis of expanded gene families in tetraploid *D. nipponica* (hapA–hapD).
**Table S15.** KEGG pathway analysis of expanded gene families in tetraploid *D. nipponica* (hapA–hapD).
**Table S16.** Allelic distribution across chromosomes in the tetraploid *D. nipponica* genome.
**Table S17.** Allele‐specific expression within the diosgenin biosynthetic pathway in the tetraploid *D. nipponica*.
**Table S18.** Tissue‐dependent allele‐specific expression patterns of diosgenin biosynthetic genes in autotetraploid *D. nipponica*.
**Table S19.** Relative quantification of metabolites in diploid and autotetraploid *D. nipponica*.
**Table S20.** Metabolites exhibiting significant differential abundance between diploid and tetraploid *D. nipponica*.
**Table S21.** Genes with positive dosage effects identified in tetraploid leaves.
**Table S22.** Genes with positive dosage effects identified in tetraploid stems.
**Table S23.** Genes with positive dosage effects identified in tetraploid rhizomes.
**Table S24.** Genes showing negative dosage effects in tetraploid leaves.
**Table S25.** Genes showing negative dosage effects in tetraploid stems.
**Table S26.** Genes showing negative dosage effects in tetraploid rhizomes.
**Table S27.** GO enrichment analysis of positive dosage‐effect genes in tetraploid leaves.
**Table S28.** GO enrichment analysis of negative dosage‐effect genes in tetraploid leaves.
**Table S29.** GO enrichment analysis of positive dosage‐effect genes in tetraploid stems.
**Table S30.** GO enrichment analysis of negative dosage‐effect genes in tetraploid stems.
**Table S31.** GO enrichment analysis of positive dosage‐effect genes in tetraploid rhizomes.
**Table S32.** GO enrichment analysis of negative dosage‐effect genes in tetraploid rhizomes.
**Table S33.** Flavonoid biosynthetic genes in the diploid and tetraploid *D. nipponica* (hapA–hapD).
**Table S34.** Positive and negative dosage effect genes related to diosgenin biosynthesis in autotetraploid *D. nipponica*.
**Table S35.** Expression of transcriptionally active alleles of flavonoid biosynthetic genes in autotetraploid *D. nipponica*.
**Table S36.** Dosage‐ and negative dosage‐effect genes related to flavonoid biosynthesis in autotetraploid *D. nipponica*.
**Table S37.** Geographic distribution and ploidy level of *D. nipponica* populations used in this study.
**Table S38.** Demographic parameter estimates inferred by fastsimcoal2 for diploid and autotetraploid *D. nipponica*.
**Table S39.** List of 155 candidate selective genes and their functional classification.
**Table S40.** Summary statistics of Illumina sequencing data.

## Data Availability

The data that support the findings of this study are openly available in Figshare at https://figshare.com/s/66604fcd8a78b418a74e.
