# Supplementary material for: Haplotype‐resolved autotetraploid genome of Dioscorea nipponica provides insights into recent polyploidization, metabolic rewiring, and environmental adaptation
Source: Plant J. 2026 Sep 28;127(6):e71153. doi: 10.1111/tpj.71153 (PMC13618807; doi:10.1111/tpj.71153)
Supplement: Supplementary file 1 [file TPJ-127-0-s002.pdf]

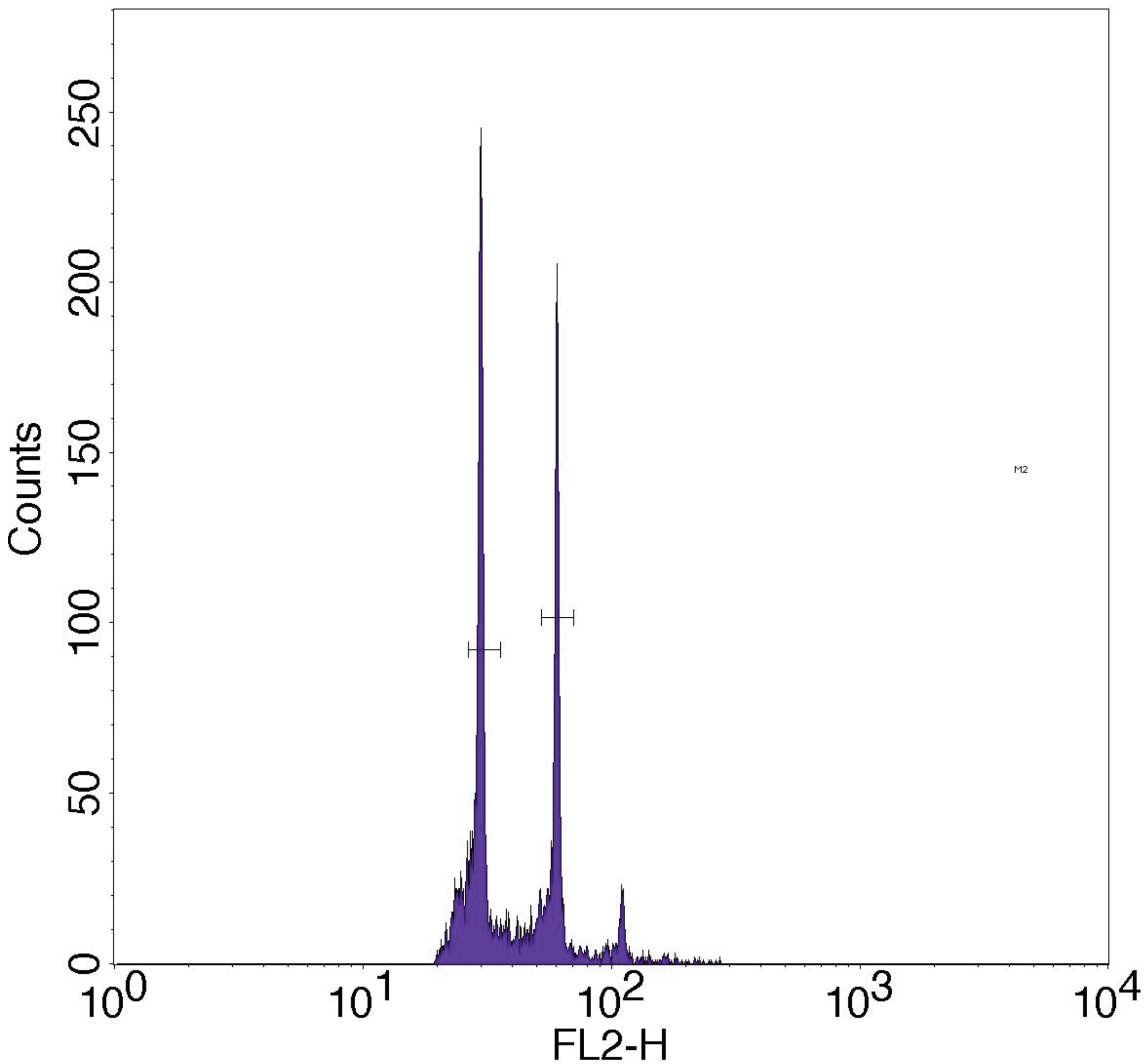

Counts
0
50
100
150
200
250
M2
10^0
10^1
10^2
10^3
10^4
FL2-H

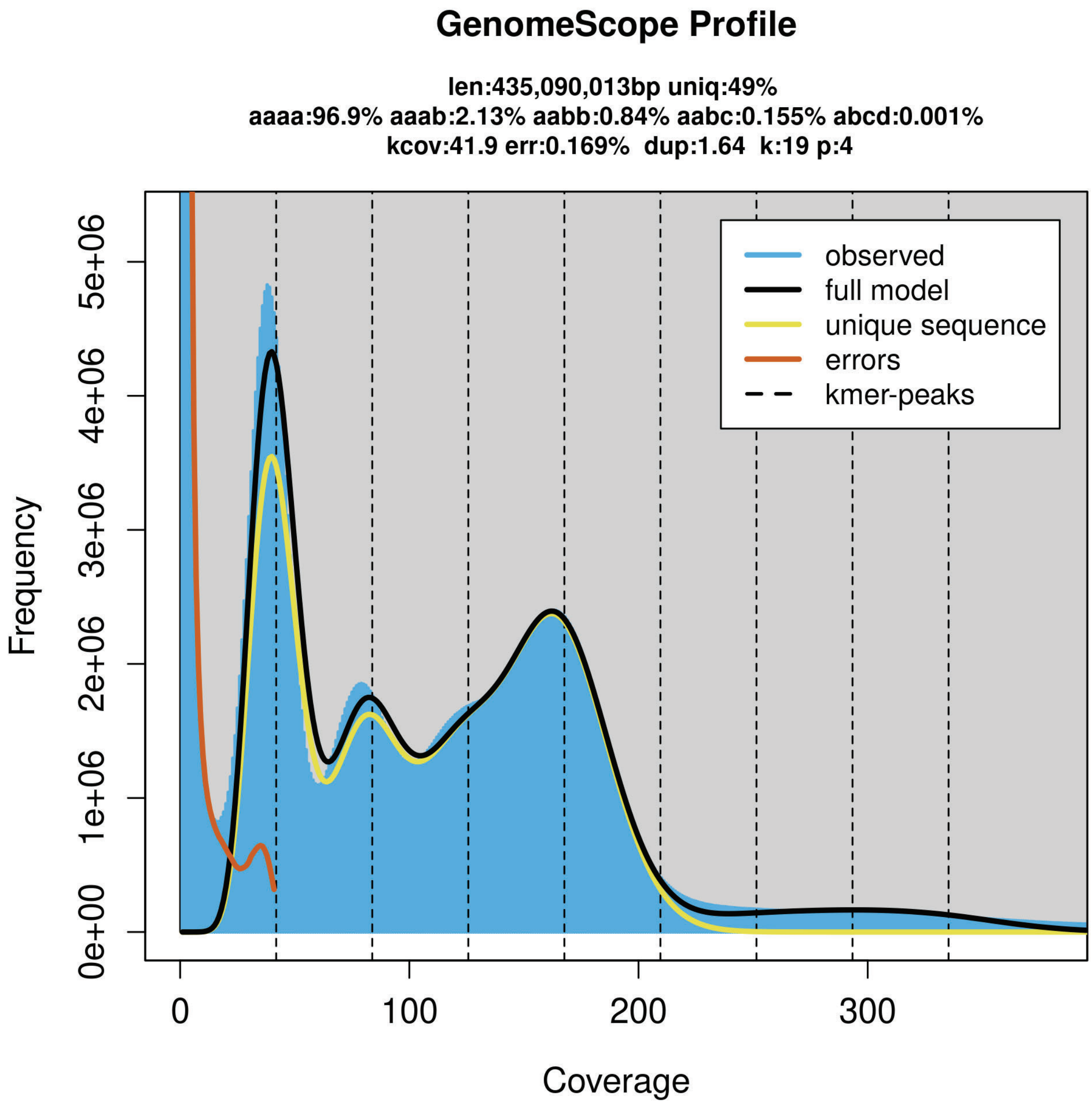
GenomeScope Profile
len:435,090,013bp uniq:49%
aaaa:96.9% aaab:2.13% aabb:0.84% aabc:0.155% abcd:0.001%
kcov:41.9 err:0.169% dup:1.64 k:19 p:4
observed
full model
unique sequence
errors
kmer-peaks
Frequency
0e+00
1e+06
2e+06
3e+06
4e+06
5e+06
0
100
200
300
Coverage

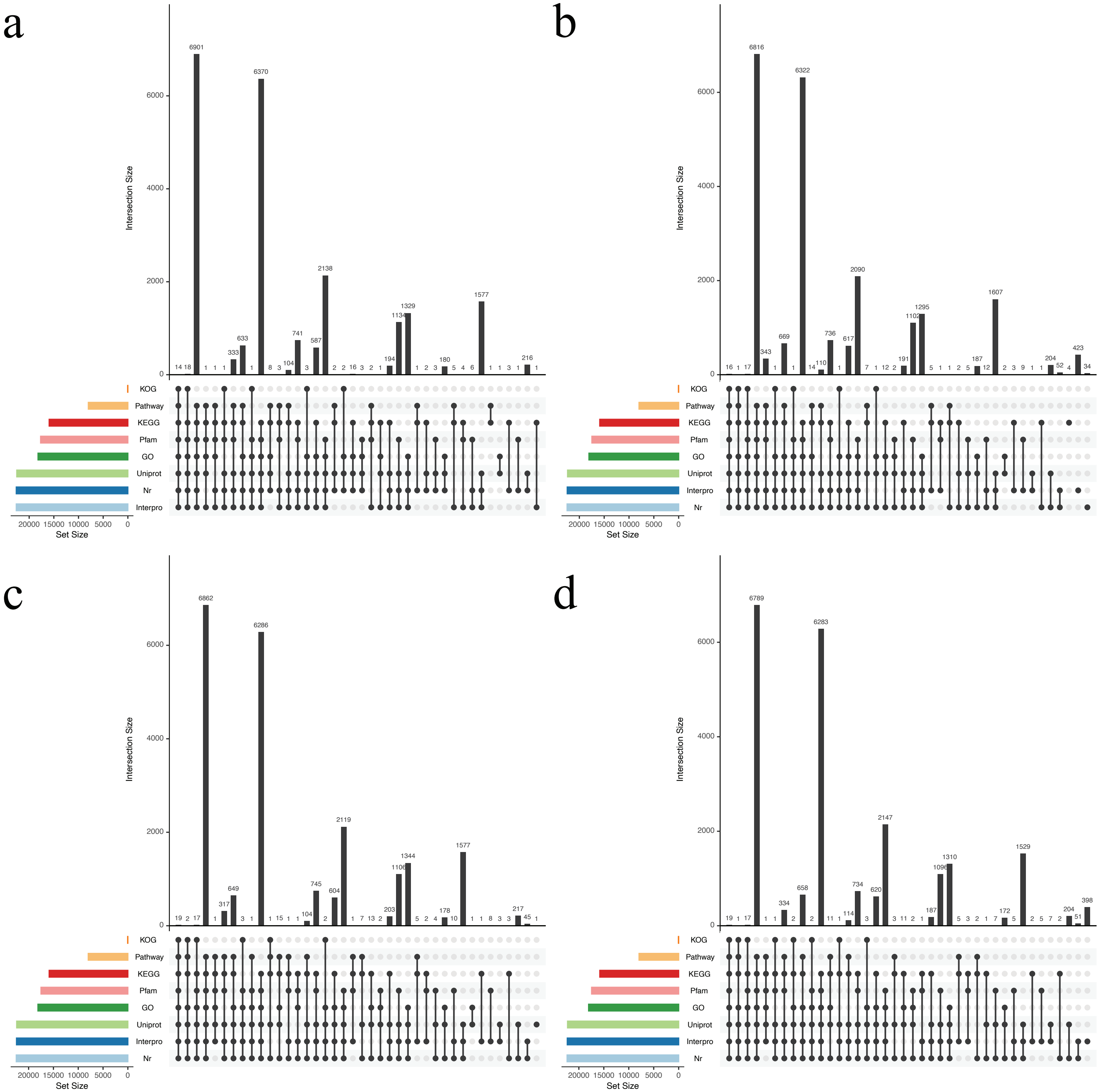
a
b
c
d
Intersection Size
Set Size
KOG
Pathway
KEGG
Pfam
GO
Uniprot
Nr
Interpro
6901
6370
2138
1577
1329
1134
741
633
587
333
216
194
180
104
6816
6322
2090
1607
1295
1102
736
669
617
423
343
204
191
187
110
6862
6286
2119
1577
1344
1106
745
649
604
317
217
203
178
104
6789
6283
2147
1529
1310
1096
734
658
620
398
334
204
187
172
114
0
2000
4000
6000
20000
15000
10000
5000

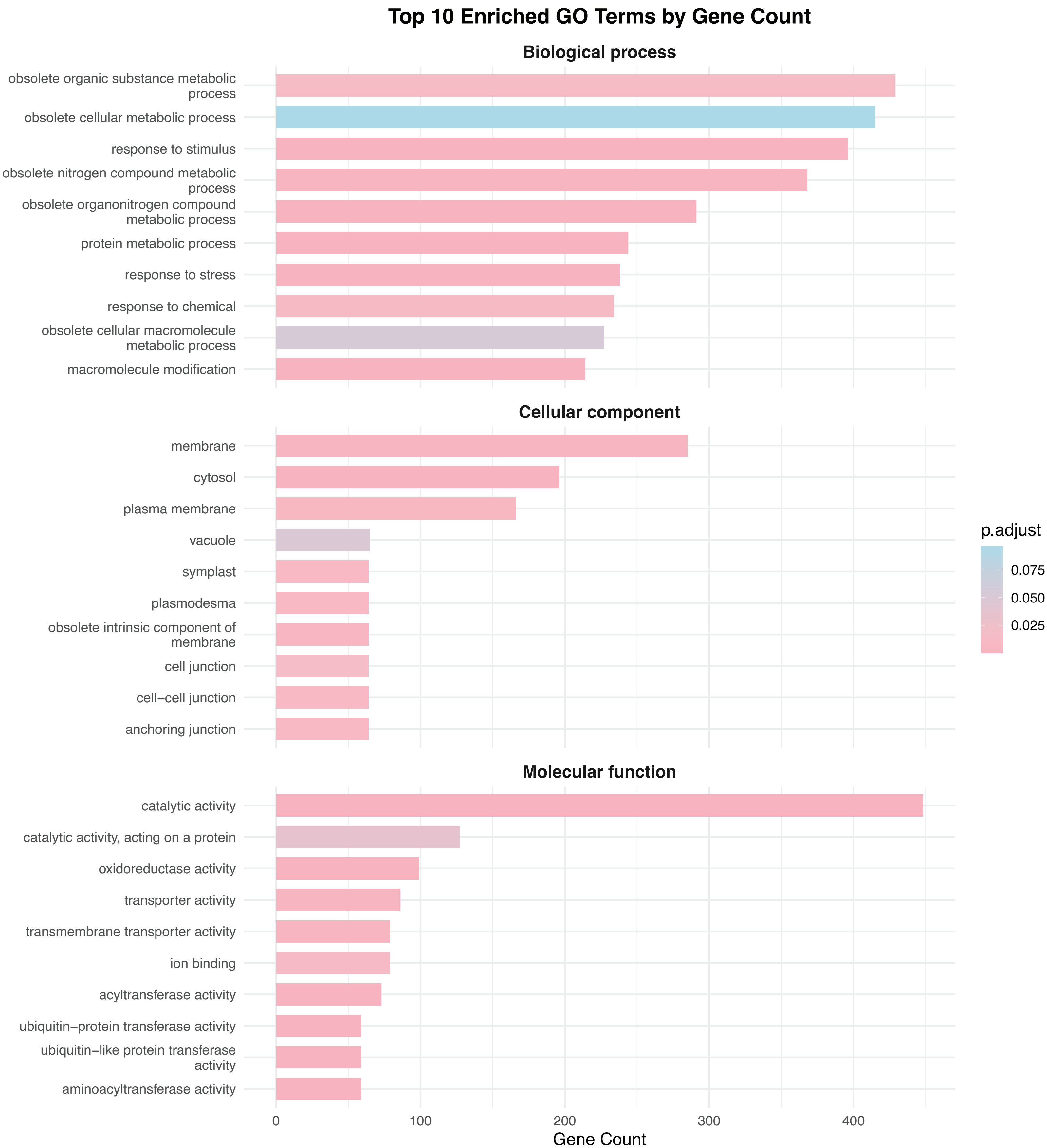
Top 10 Enriched GO Terms by Gene Count
Biological process
obsolete organic substance metabolic process
obsolete cellular metabolic process
response to stimulus
obsolete nitrogen compound metabolic process
obsolete organonitrogen compound metabolic process
protein metabolic process
response to stress
response to chemical
obsolete cellular macromolecule metabolic process
macromolecule modification
Cellular component
membrane
cytosol
plasma membrane
vacuole
symplast
plasmodesma
obsolete intrinsic component of membrane
cell junction
cell−cell junction
anchoring junction
Molecular function
catalytic activity
catalytic activity, acting on a protein
oxidoreductase activity
transporter activity
transmembrane transporter activity
ion binding
acyltransferase activity
ubiquitin−protein transferase activity
ubiquitin−like protein transferase activity
aminoacyltransferase activity
0
100
200
300
400
Gene Count
p.adjust
0.075
0.050
0.025

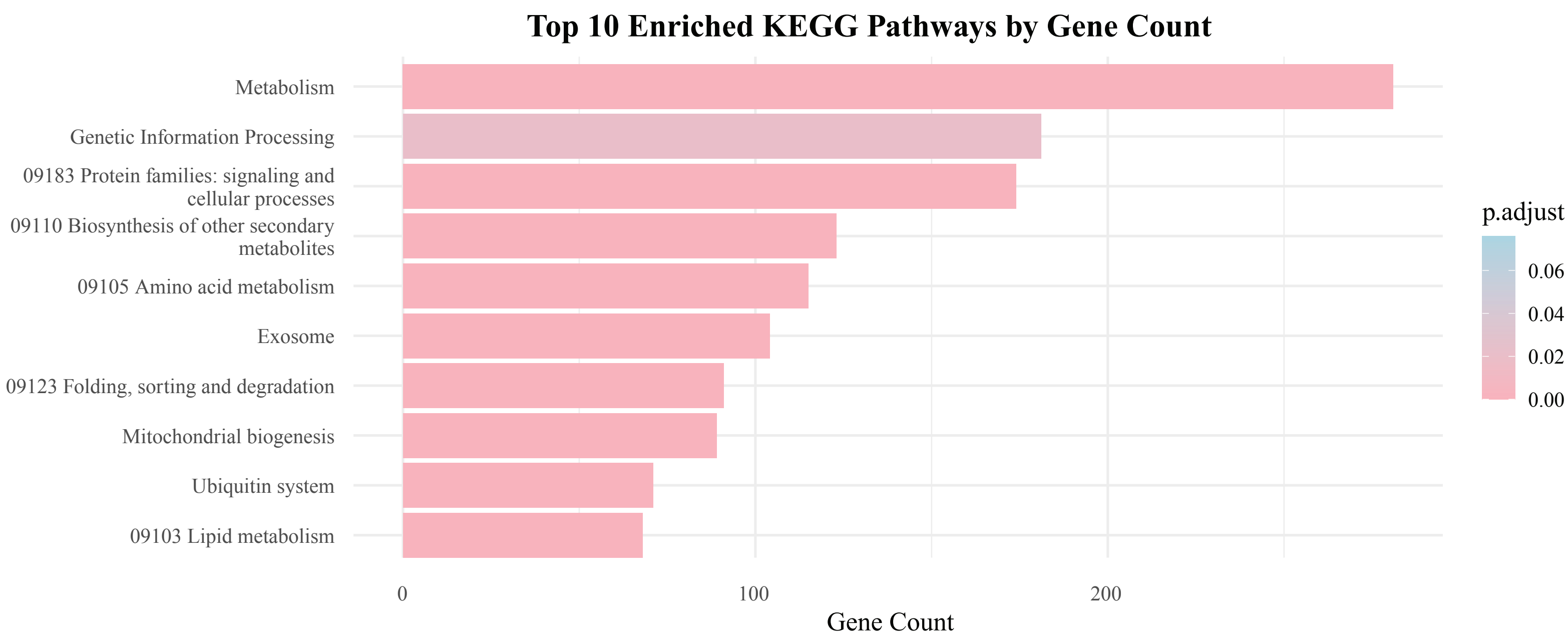
Top 10 Enriched KEGG Pathways by Gene Count
Metabolism
Genetic Information Processing
09183 Protein families: signaling and cellular processes
09110 Biosynthesis of other secondary metabolites
09105 Amino acid metabolism
Exosome
09123 Folding, sorting and degradation
Mitochondrial biogenesis
Ubiquitin system
09103 Lipid metabolism
0
100
200
Gene Count
p.adjust
0.06
0.04
0.02
0.00

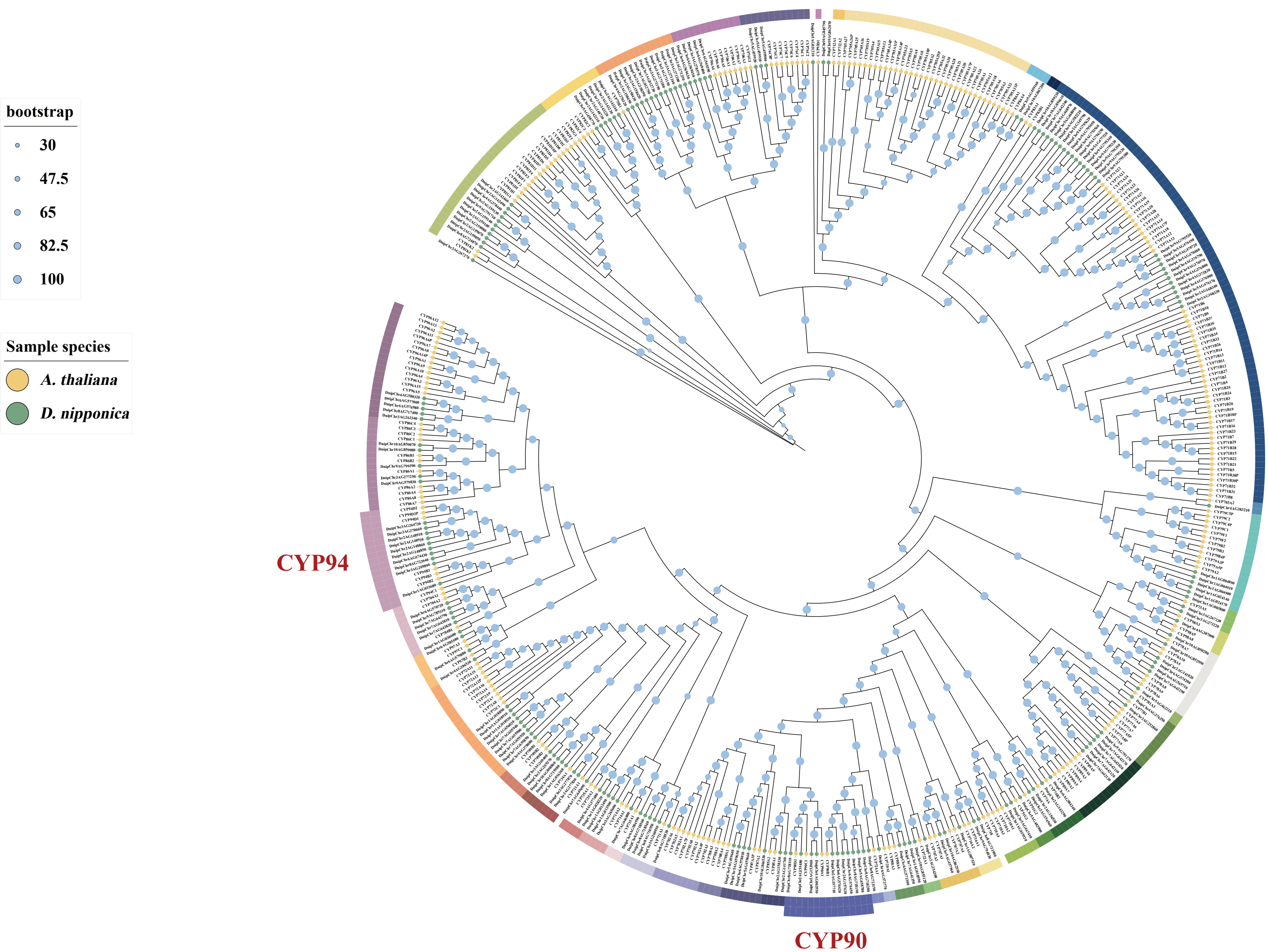
bootstrap
30
47.5
65
82.5
100
Sample species
A. thaliana
D. nipponica
CYP94
CYP90

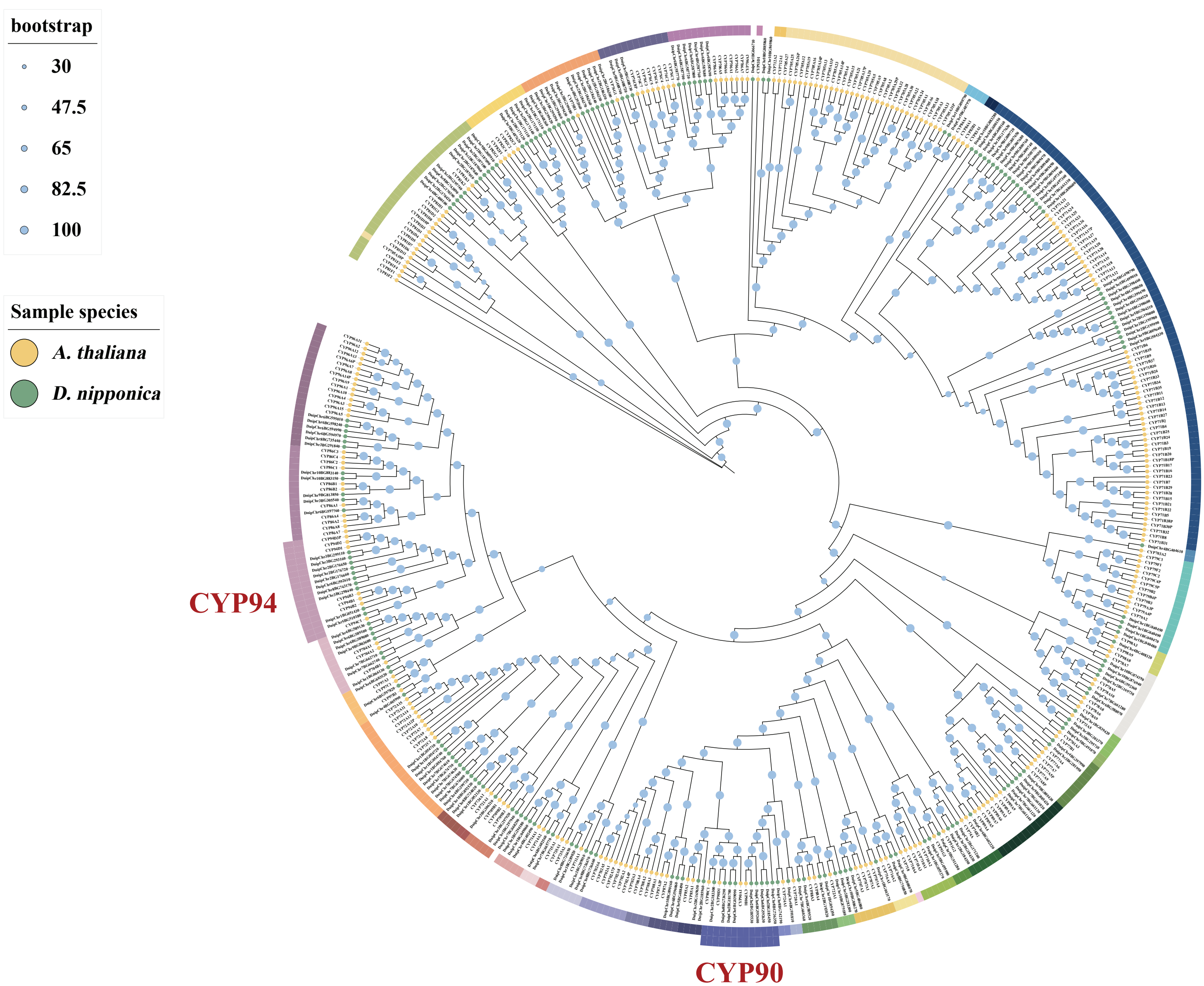
bootstrap
30
47.5
65
82.5
100
Sample species
A. thaliana
D. nipponica
CYP94
CYP90

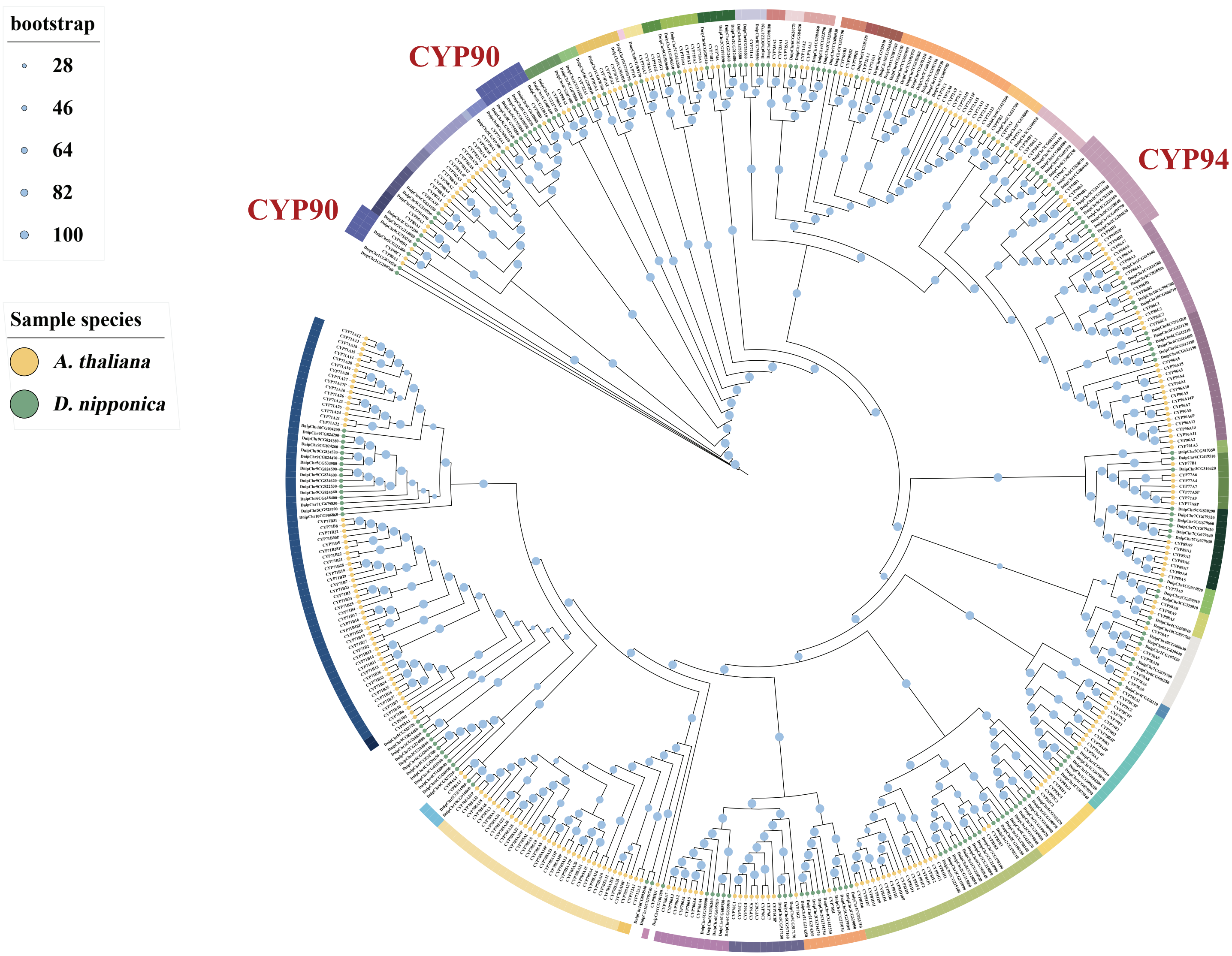

bootstrap
28
46
64
82
100
Sample species
A. thaliana
D. nipponica
CYP90
CYP90
CYP94

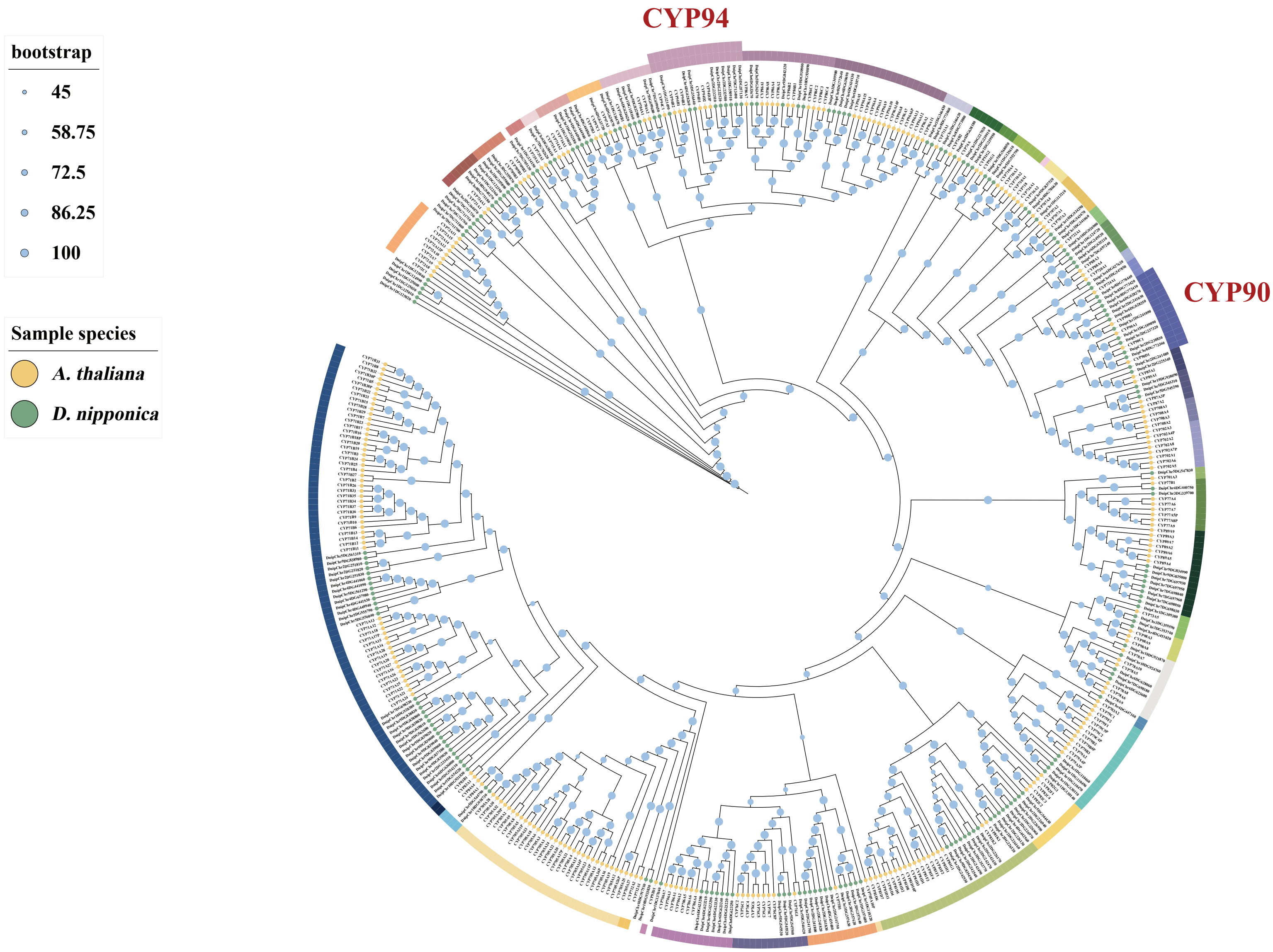
CYP94
CYP90
bootstrap
45
58.75
72.5
86.25
100
Sample species
A. thaliana
D. nipponica

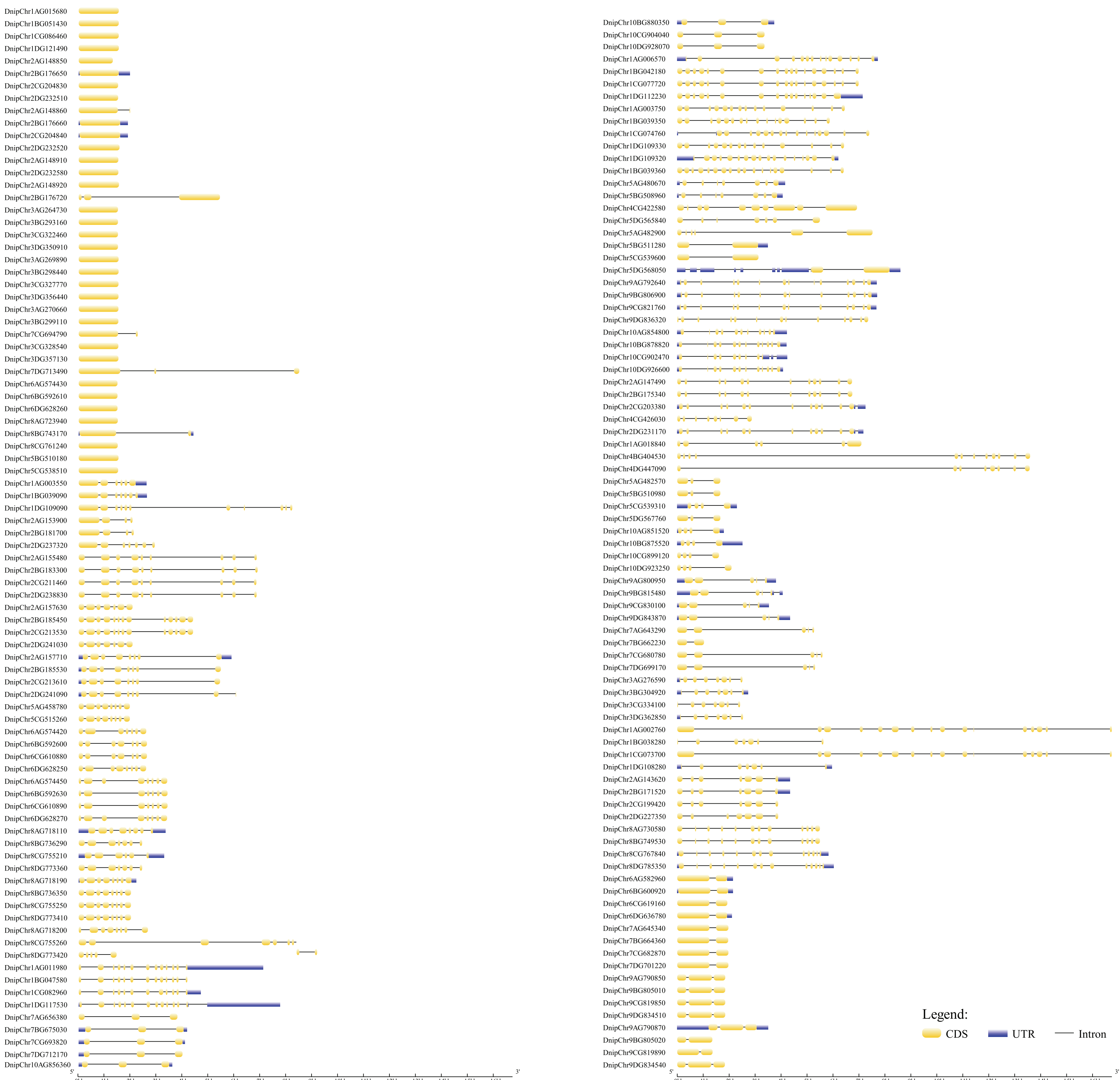
DnipChr1AG015680
DnipChr1BG051430
DnipChr1CG086460
DnipChr1DG121490
DnipChr2AG148850
DnipChr2BG176650
DnipChr2CG204830
DnipChr2DG232510
DnipChr2AG148860
DnipChr2BG176660
DnipChr2CG204840
DnipChr2DG232520
DnipChr2AG148910
DnipChr2DG232580
DnipChr2AG148920
DnipChr2BG176720
DnipChr3AG264730
DnipChr3BG293160
DnipChr3CG322460
DnipChr3DG350910
DnipChr3AG269890
DnipChr3BG298440
DnipChr3CG327770
DnipChr3DG356440
DnipChr3AG270660
DnipChr3BG299110
DnipChr7CG694790
DnipChr3CG328540
DnipChr3DG357130
DnipChr7DG713490
DnipChr6AG574430
DnipChr6BG592610
DnipChr6DG628260
DnipChr8AG723940
DnipChr8BG743170
DnipChr8CG761240
DnipChr5BG510180
DnipChr5CG538510
DnipChr1AG003550
DnipChr1BG039090
DnipChr1DG109090
DnipChr2AG153900
DnipChr2BG181700
DnipChr2DG237320
DnipChr2AG155480
DnipChr2BG183300
DnipChr2CG211460
DnipChr2DG238830
DnipChr2AG157630
DnipChr2BG185450
DnipChr2CG213530
DnipChr2DG241030
DnipChr2AG157710
DnipChr2BG185530
DnipChr2CG213610
DnipChr2DG241090
DnipChr5AG458780
DnipChr5CG515260
DnipChr6AG574420
DnipChr6BG592600
DnipChr6CG610880
DnipChr6DG628250
DnipChr6AG574450
DnipChr6BG592630
DnipChr6CG610890
DnipChr6DG628270
DnipChr8AG718110
DnipChr8BG736290
DnipChr8CG755210
DnipChr8DG773360
DnipChr8AG718190
DnipChr8BG736350
DnipChr8CG755250
DnipChr8DG773410
DnipChr8AG718200
DnipChr8CG755260
DnipChr8DG773420
DnipChr1AG011980
DnipChr1BG047580
DnipChr1CG082960
DnipChr1DG117530
DnipChr7AG656380
DnipChr7BG675030
DnipChr7CG693820
DnipChr7DG712170
DnipChr10AG856360
5'
3'
0kb 1kb 2kb 3kb 4kb 5kb 6kb 7kb 8kb 9kb 10kb 11kb 12kb 13kb 14kb 15kb 16kb
DnipChr10BG880350
DnipChr10CG904040
DnipChr10DG928070
DnipChr1AG006570
DnipChr1BG042180
DnipChr1CG077720
DnipChr1DG112230
DnipChr1AG003750
DnipChr1BG039350
DnipChr1CG074760
DnipChr1DG109330
DnipChr1DG109320
DnipChr1BG039360
DnipChr5AG480670
DnipChr5BG508960
DnipChr4CG422580
DnipChr5DG565840
DnipChr5AG482900
DnipChr5BG511280
DnipChr5CG539600
DnipChr5DG568050
DnipChr9AG792640
DnipChr9BG806900
DnipChr9CG821760
DnipChr9DG836320
DnipChr10AG854800
DnipChr10BG878820
DnipChr10CG902470
DnipChr10DG926600
DnipChr2AG147490
DnipChr2BG175340
DnipChr2CG203380
DnipChr4CG426030
DnipChr2DG231170
DnipChr1AG018840
DnipChr4BG404530
DnipChr4DG447090
DnipChr5AG482570
DnipChr5BG510980
DnipChr5CG539310
DnipChr5DG567760
DnipChr10AG851520
DnipChr10BG875520
DnipChr10CG899120
DnipChr10DG923250
DnipChr9AG800950
DnipChr9BG815480
DnipChr9CG830100
DnipChr9DG843870
DnipChr7AG643290
DnipChr7BG662230
DnipChr7CG680780
DnipChr7DG699170
DnipChr3AG276590
DnipChr3BG304920
DnipChr3CG334100
DnipChr3DG362850
DnipChr1AG002760
DnipChr1BG038280
DnipChr1CG073700
DnipChr1DG108280
DnipChr2AG143620
DnipChr2BG171520
DnipChr2CG199420
DnipChr2DG227350
DnipChr8AG730580
DnipChr8BG749530
DnipChr8CG767840
DnipChr8DG785350
DnipChr6AG582960
DnipChr6BG600920
DnipChr6CG619160
DnipChr6DG636780
DnipChr7AG645340
DnipChr7BG664360
DnipChr7CG682870
DnipChr7DG701220
DnipChr9AG790850
DnipChr9BG805010
DnipChr9CG819850
DnipChr9DG834510
DnipChr9AG790870
DnipChr9BG805020
DnipChr9CG819890
DnipChr9DG834540
5'
3'
0kb 1kb 2kb 3kb 4kb 5kb 6kb 7kb 8kb 9kb 10kb 11kb 12kb 13kb 14kb 15kb 16kb
Legend:
CDS
UTR
Intron

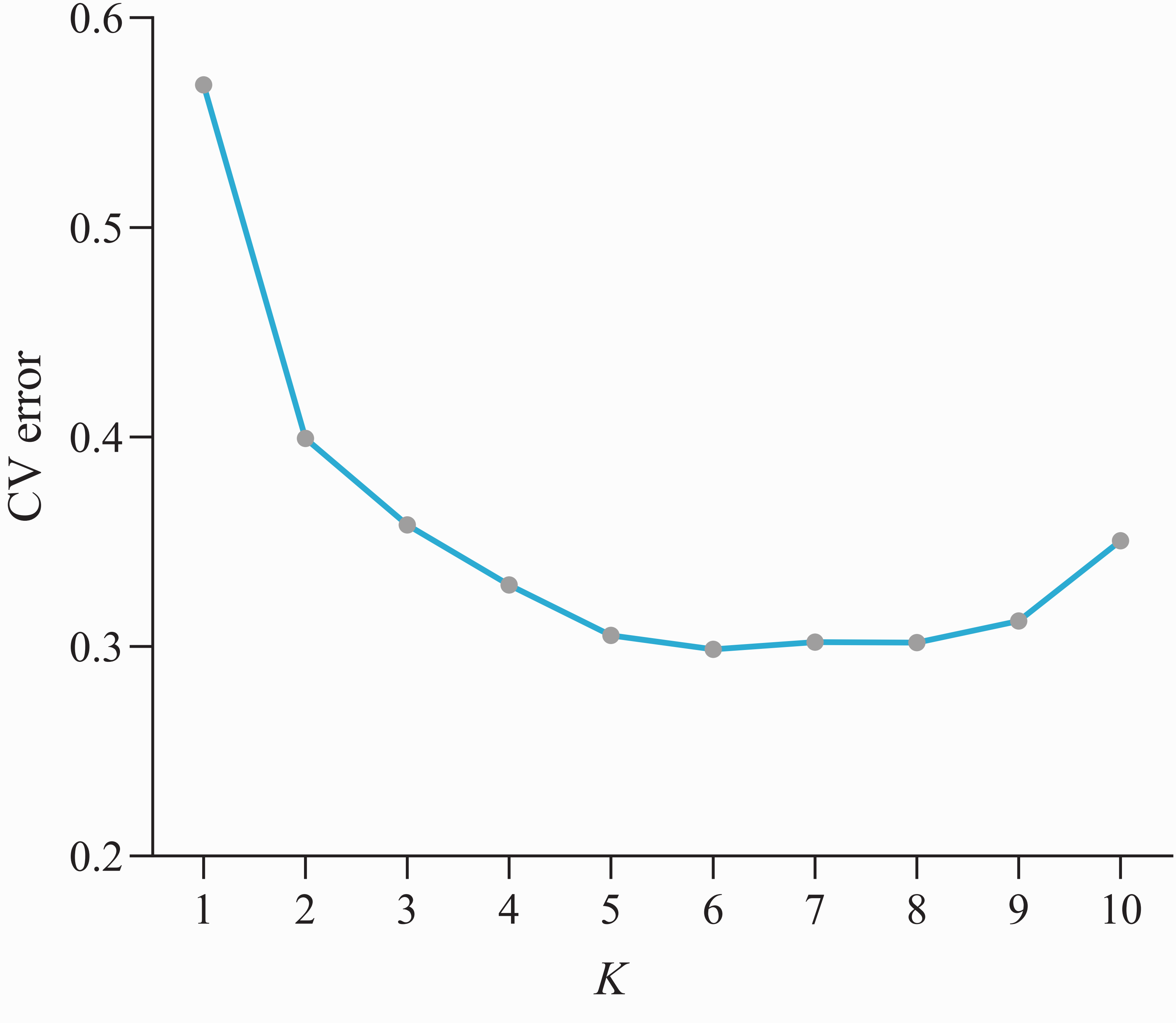
0.6
0.5
0.4
0.3
0.2
CV error
1
2
3
4
5
6
7
8
9
10
K
